# Urban nutrition situation in the slums of three cities in Asia during the COVID‐19 pandemic

**DOI:** 10.1111/mcn.13543

**Published:** 2023-10-09

**Authors:** Carolyn I. Auma, Rebecca Pradeilles, Heather Ohly, Sabrina Eymard‐Duvernay, Kristian A. Brizendine, Jessica Blankenship, Anusara Singhkumarwong, Sophie Goudet

**Affiliations:** ^1^ School of Food Science and Nutrition University of Leeds Leeds UK; ^2^ UMR MoISA (Montpellier Interdisciplinary Centre on Sustainable Agri‐Food Systems), (Univ Montpellier, CIRAD, CIHEAM‐IAMM, INRAE, Institut Agro, IRD) Montpellier France; ^3^ Nutrition Research, Dikoda London UK; ^4^ Nutrition Section UNICEF East Asia and the Pacific Regional Office Bangkok Thailand; ^5^ Nutrition Section, WFP Asia and Pacific Regional Office Bangkok Thailand; ^6^ Centre for Global Health and Human Development, School of Sport, Exercise and Health Sciences Loughborough University Loughborough UK

**Keywords:** COVID‐19, food insecurity, Jakarta, Quezon City, urban poor, Yangon

## Abstract

Urban‐poor households are disproportionately food insecure. The Status and Determinants of Food Insecurity and Undernutrition in Poor Urban Settings (SDFU) cross‐sectional surveys were conducted in 2020–2021 to assess the impacts of COVID‐19 on food security and diet quality among urban poor women of reproductive age (WRA) and children under 5 (CU5) in Jakarta, Quezon City, and Yangon. Data, collected on food insecurity and child and maternal diet quality using Computer Assisted Telephone Interviewing (CATI), were compared with prepandemic surveys. Prevalence for food insecurity and diet quality indicators were computed. Eight in 10 households in all three cities reported reduced incomes, with 6 in 10 worried about food the previous year. Over 10% of households in all cities substituted nutrient‐dense (ND) foods with cheaper alternatives; yet less than 50% of children 6–59 months ate sugar‐sweetened beverages or sweet and savoury snacks. Compared with baseline, women's minimum dietary diversity (MDD) in the three cities was significantly lower (up to 30% lower in Yangon and Jakarta), while the prevalence of children (6–23 months) meeting MDD was lower by 17.4%–42.5% in all cities. MDD was attained by >40% of children (24–59 months) in Yangon and Jakarta but only 12.6% in Quezon City. To improve food security and diet quality, multi‐sectoral interventions are needed, including distributing ND foods and cash assistance to vulnerable households with CU5 and WRA and delivering targeted nutrition training to encourage appropriate complementary feeding practices and purchasing and consumption of ND foods.

## Key messages

- Food insecurity levels were higher compared with baseline. More than 60% of all surveyed households across all three cities worried about food 12 months before the survey.
- Due to financial stress, at least 10% of households across the three cities substituted ND foods, e.g., meat, poultry, fish, fruit, vegetables, beans, pulses, and tofu, with cheaper, nutrient‐poor alternatives, although this proportion varied by city (16%‐75% of surveyed households).
- Compared with baseline, a lower proportion of women and children 6–59 months achieved MDD, indicating poorer diet quality during the pandemic.
- Sugar‐sweetened beverages and sweet and savoury snacks intakes were lower than baseline among children 6–59 months.

## Graphical abstract

During the pandemic, many urban‐poor households in Myanmar, Indonesia, and the Philippines experienced moderate or severe food insecurity. Of the three populations, urban‐poor households in the Philippines were most food insecure, with at least 8 in 10 households worried about food the year before the pandemic, and at least 6 in 10 households food insecure.

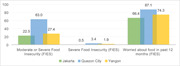

## 1 INTRODUCTION

Although half of all Asians already reside in urban areas, over 65% of the Asian population will live in urban areas by 2050 owing to rapid urbanisation that is expected to take place in many low‐ and middle‐income countries (United Nations Department of Economic and Social Affairs, 2018; World Bank, 2020a). Many Asian countries have already seen an increase in the number of both, larger and smaller cities, in recent years. In these countries, urbanisation is taking place amidst economic growth and poor urban planning and governance, resulting in many urbanites residing in informal settlements and slums, which are characterised by housing that lack adequate, safe and reliable water and sanitation facilities, are restricted in space and are constructed of materials that are neither durable nor protective, moreover lacking tenure (UN‐HABITAT United Nations Human Settlement Programme, 2006, 2018). In Asia, 6 in 10 people are expected to live in slums by 2025, 20% more than the global estimate (UN‐HABITAT United Nations Human Settlement Programme, 2018). Moreover, in the same period, the number of slum dwellers in Southeast Asia, alone, is expected to reach 476 million (UN‐HABITAT United Nations Human Settlement Programme, 2018). Jakarta (Indonesia), Quezon City (Philippines) and Yangon (Myanmar) are three large Asian cities that have experienced rapid urbanisation in recent years and have large slum populations (World Food Programme, 2021a, 2021b, 2021c). In these cities, as in many such cities in low and middle‐income countries, the urban poor, whose lives are characterised by high levels of poverty and socioeconomic inequality, predominantly reside under slum conditions.

There is a widespread notion that, compared with people living in rural areas, those living in urban areas face fewer food security challenges, particularly those concerning physical food access (Vilar‐Compte et al., 2021). What is clear, however, is that increased physical ‘food access’ in urban areas is not a blanket advantage experienced by all urban dwellers—the urban poor are often at a disadvantage. Moreover, urban averages often overlook informal settlements that are not captured in formal surveys, thereby masking the large disparities between urban poor and wealthier households. The urban poor face disproportionately higher food insecurity and are, therefore, at a higher risk of undernutrition than even their rural counterparts who often have access to farmland for their own production, which the urban poor lack (Chatterjee et al., 2012; Onyango et al., 2021; Piaseu & Mitchell, 2004). Food insecurity among the urban poor primarily stems from income poverty, which reduces individual and household purchasing power (Kimani‐Murage et al., 2014; Tacoli, 2019). If the cost of a nutritious diet is high, it will be unattainable for the urban poor, whether at certain periods of time or consistently. In urban areas, energy‐dense, nutrient‐poor ultraprocessed foods and sugar‐sweetened drinks, are often cheaper, tasty and widely available. The urban poor, being unable to afford nutritious food, may be forced to depend on such foods, which are usually high in saturated fat, trans‐fat, and refined carbohydrates, and low in dietary fibre and other key nutrients (Vilar‐Compte et al., 2021). Such foods, although low in nutritional value, require minimal preparation, which suits many low‐income slum households as they often lack adequate heating, cooking tools, safe water and space (UN‐HABITAT United Nations Human Settlement Programme, 2006; Vilar‐Compte et al., 2021). When external shocks, for example, illness affect the breadwinner, urban poor households are at increased vulnerability for food insecurity as they lack access to the more traditional safety nets in rural areas, such as own food production, while at the same time facing challenges such as restricted market access (Akseer et al., 2020; Tacoli, 2019). The recent COVID‐19 pandemic is one extreme example of a global external shocks with serious repercussions for urban poor populations. Beginning in early 2020, the pandemic was managed by governments in Southeast Asia via several control measures, leading to disrupted movements, livelihoods, food systems, and nutrition‐related services. These measures dramatically impacted food supply chains, food security, and household income throughout Southeast Asia with negative impacts well documented in Indonesia, the Philippines and Myanmar (Headey et al., 2020; International Food Policy Research Institute, 2021; World Food Programme, 2020, 2022). These negative impacts of the pandemic on food security pose a threat to gains made towards achieving the 2030 Sustainable Development Goals (SDGs), such as SDG2 (zero hunger by 2030) and World Health Assembly goals to reduce national‐level stunting, low birthweight and anaemia. Moreover, effects could be even more detrimental in a context like Myanmar, which for decades has suffered political unrest that has affected the country's economy, resulting in high poverty even before the pandemic.

Against this background, four agencies, the Food and Agriculture Organization of the United Nations (FAO), the World Food Programme (WFP), the World Health Organization (WHO), and the United Nations Children's Emergency Fund (UNICEF), conducted the Status and Determinants of Food Insecurity and Undernutrition in Urban Areas (SDFU) surveys in the three cities of Jakarta (Indonesia), Quezon City (the Philippines) and Yangon (Myanmar). The surveys aimed to assess the impact of the COVID‐19 pandemic on food security and diet quality among women and children residing in urban poor areas of Jakarta, Quezon City and Yangon by comparing changes in selected indicators.

## 2 METHODS

The Status and Determinants of Food Insecurity and Undernutrition in Poor Urban Settings (SDFU) surveys were initiated under a joint FAO, UNICEF and WFP initiative to develop an adapted approach to survey poor urban populations living in slums. Adaptation to the urban settings was made in various ways including engagement with city stakeholders, targeting and sampling respondents among poor urban communities, addition of specific urban‐focused questions/modules (e.g., food consumption away from home, coping strategies that are possible in cities). The SDFU was meant to be implemented face to face, however, due to the COVID‐19 pandemic, the SDFUs in 2020 and 2021 were collected remotely. The study followed a two‐step approach: (1) first the baseline surveys were identified from different sources for comparison purposes, (2) then the endline surveys were conducted during the COVID‐19 pandemic.

### 2.1 Surveys for baseline comparisons

Survey data were compared with pre‐COVID‐19 data. In each country, the baseline data were carefully selected from the most comparable data from poor urban communities given important data gaps; the Status and Determinants of Food Insecurity and Undernutrition in Poor Urban Settings, Myanmar in 2018 (SDFUM 2018) for Yangon, the UNICEF 2018 Reaching Every Community (REC) trial for Jakarta and multiple survey sources for Quezon City (*n* = 413), five urban areas (Manila, Cebu, Zamboanga, Naga and Iloilo) (Rohner et al., 2013) (*n* = 1784), DHS 2017 urban (Philippine Statistics Authority [PSA] and ICF 2018). In Jakarta and Yangon, the SDFUs took place in similar locations among similar populations whereas for Quezon City, the baseline data were formed from various surveys to have data for most indicators. Comparisons with these baselines are indicative and no statistical analyses were possible between baseline and SDFUs surveys.

#### 2.1.1 Yangon

For baseline comparisons, data from the SDFUM2018 were used. The SDFUM2018 was conducted in 2018 among 3000 households with and without CU5 in formal and informal urban poor settlements in 11 townships in Yangon.

#### 2.1.2 Quezon City

For baseline comparisons, data from a previous similar survey were used dated 2013 and 2017. SDFUP was implemented via telephone interviews by the University of the Philippines Los Baños (UPLB), a public university in the Philippines.

#### 2.1.3 Jakarta

For baseline comparisons, available data from the 2018 UNICEF REC trial, a comprehensive evaluation of immunization interventions in Jakarta's urban slums, were used.

### 2.2 Sampling, sample size and study site for endline surveys

Data at endline were collected remotely from selected urban slum households in Jakarta, Quezon City and Yangon using Computer Assisted Telephone Interviewing (CATI), following survey piloting and questionnaire revision. Full sampling details, including sample size, can be found in Supporting Information 1 and are highlighted below.

#### 2.2.1 Yangon

In Yangon, the survey (SDFUM2021) was carried out in August–September 2021 among poor formal settlements in the peri‐urban townships within the same locations as SDUM2018. Based on Infant and Young Child Feeding (IYCF) indicators and malnutrition prevalence from SDFUM2018, a sample size of 2300 children under 5 (U5) was deemed necessary to ensure representativeness for SDFUM2021. Households with children U5 were oversampled, aiming for a sample size of 3300 households, including 3300 households with children U5 (CU5) and 1000 households with children under 2 (CU2). For SDFUM2021, attempts were made to contact all households that participated in the SDFUM 2018 baseline survey. To reach the required sample size, snowball sampling was used, with additional sampling lists obtained from partners operational in Yangon, such as Terre des Hommes, Myanmar Health Assistant Association, WFP Urban Response, and World Vision International. In all, 3077 households with children (*n* = 2201) and without children (*n* = 876) were sampled. Data collection for SDFUM 2021 was implemented by the WFP in‐house enumerators (*n* = 40) who had previous experience in data collection from the 2020–2021 Maternal and Child Cash Transfer (MCCT) baseline surveys in Shan State and Ayeyarwady Region.

#### 2.2.2 Quezon City

The survey (SDFUP2020) took place in October–December 2020 in selected barangays (smallest administrative division) in six districts. Sample size (*n* = 3000) was first determined to provide estimates of acceptable precision for children stunting prevalence, that is, 1500 households with CU2 and 1500 households with CU5. The sampling frame details were taken from the Quezon City Health Office, particularly the health centres covering depressed and slum areas in the six survey districts. However, since the SDFUP2020 was conducted remotely, sampled households included only those with CU5 in which a mobile phone number was available. Households were sampled randomly within each district, with sampling stratified depending on the total number of eligible households per district yielding a final sample of 2474 total households and 2667 CU5.

#### 2.2.3 Jakarta

In Jakarta, the SDFUI2020 took place in September–October 2020. Potential survey sites were predetermined and identified as the 23 UNICEF intervention communities and 23 control communities in three areas (Jakarta Barat, Jakarta Timur, and Jakarta Utara). Of these, cadres from 19 communities responded provided a list of contacts which became the study sites for the SDFUI2020. Standardized sample size estimation methodology determined the minimum sample size (730 households in total and 584 households with CU5) needed for province‐level representation. Sampling followed a two‐stage cluster survey with a hamlet serving as a cluster. A final sample size of 777 households with CU5 was achieved. SDFUI 2020 was implemented by Reconstra, an Indonesian research company, which led the UNICEF 2018 REC trial (baseline survey).

### 2.3 Data collection questionnaires for endline surveys

In all three locations, questionnaires captured data on children's and mother's dietary intakes and relevant associated variables at the household level. The questionnaires were built upon the Multiple Indicator Cluster Survey (MICS) modules. The Household questionnaire, an example of which can be found in Supporting Information 1 included data on (i) household profile, (ii) employment situation, conditions and remittance, (iii) Expenditure, (iv) credit or debt, remittances, (v) housing, (vi) food consumption score and market food access, (vii) coping strategies, (viii) household food insecurity access scale and household food provisioning, (ix) water, sanitation and hygiene, (x) health, and (xi) COVID‐19 shock. The Mother and Child questionnaire, an example of which can be found in Supporting Information 1 captured data on (i) children and mother profile (e.g., age, education for mother only), (ii) women's dietary diversity and food purchased for women, (iii) infant and young children feeding practices, (iv) child health and nutrition, including immunization, morbidity, (v) child's illness and care, (vi) food purchased for children. Data on dietary intake was collected via a qualitative 24 h recall method, that is, women were asked to recall what they ate and drank the day or night before the interview day.

#### 2.3.1 Sociodemographic variables and impact of the COVID‐19 pandemic

Households were classified into income groups based on the international poverty line, that is, extreme poverty (<US$1.90 per day), lower middle‐income households (>US$1.90 and <US$3.20 per capita, per day), upper‐middle income households (>US$3.20 and <US$5.50 per capita, per day), and nonpoor households (above all poverty lines) (>US$5.50). To assess the impact of the pandemic, the COVID‐19 Impact Index, a composite of the COVID‐19 Shock Severity category and Livelihood‐Based Coping Strategy (LBCS) category classified households in one of three COVID‐19 Impact Index levels: Low (green), Moderate (yellow), and Severe (red). The COVID‐19 Shock Severity category (little, medium, high) was determined from answers to 12 questions indicating the household respondent's concerns pertaining to COVID‐19. LBCS category (stress, crisis, emergency, none) was derived from a series of questions regarding the household's experience with livelihood stress and asset depletion in the 30 days before survey. For households taking a loan for food, water, or health, the COVID‐19 Impact Index was immediately set to ‘severe’, overriding all other factors. Details on the COVID‐19 impact index can be found in Supporting Information 2.

#### 2.3.2 Food security and diet quality and indicators

Standardised indicators were used to assess food security and child and maternal diet quality (see Supporting Information 2). Early initiation of breastfeeding (EIBF) was defined as the proportion of infants that were put on the mother's breast within an hour of birth, whereas exclusive breastfeeding (EBF) was defined as the proportion of infants that were fed on only breast milk, excluding additional water, drinks and food, within their first 6 months of life (WHO & UNICEF, 2021). Dietary data were used to calculate the indicator of minimum dietary diversity for women (MDD‐W), which provides an indication of the nutrient adequacy of a woman's diet (FAO, 2021). According to this, a woman had an adequately diverse diet if, on the previous day, she consumed foods from at least 5 out of 10 defined food groups. Energy‐dense, nutrient‐poor food consumption was assessed based on consumption of products from four food groups, that is, sugary drinks, sugar‐sweetened beverages, sweet and savoury snacks and fried foods, during the previous 24 h. To collect data on children's dietary intake, mothers were asked to recall what their children ate and drank for the 24 h period before the interview day. Dietary data were used to calculate indicators that described the adequacy of a child's diet, that is, minimum dietary diversity (MDD), minimum meal frequency (MMF), and minimum acceptable diet (MAD) (WHO & UNICEF, 2021). For children 6–23 months, MDD was defined as percent of children who consumed foods and beverages from at least five out of eight defined food groups during the previous day; MMF was defined as the percent of children who consumed solid, semisolid or soft foods (but also including milk feeds for nonbreastfed children) at least the minimum number of times during the previous day; and MAD was the percent of children who consumed a minimum acceptable diet during the previous day diet (WHO & UNICEF, 2021). The severity of food insecurity was measured using data collected with the Food Insecurity Experience Scale (FIES) module (FAO Food and Agricultural Organisation, 2016; Cafeiro et al., 2018), a set of eight questions asking household members to self‐report conditions and experiences typically associated with limited food access. Using statistical techniques based on the Rasch measurement model (FAO Food and Agricultural Organisation, 2016), data obtained were validated for internal consistency and used to produce quantitative measures along a scale of increasing severity. Based on responses to the FIES items and two globally set severity levels (thresholds), households were assigned a probability to be in one of two food security classes, that is, moderately/severely food insecure and severely food insecure.

#### 2.3.3 Household ability to engage with the market

The synthetic ‘reduced access’ indicators were constructed in three steps: (1) for each food group: construction of a ‘reduced financial access’ variable and a ‘reduced physical access’ variable (Details on the categorization can be found in Supplementary Material 2); (2) then a variable that counts how many food groups are affected by reduced access was calculated; (3) and finally, based on the association with the dependent variables in univariate, we determined the ‘x’ in the variable ‘at least x food groups are affected by reduced access’.

### 2.4 Data analysis

All analyses were conducted on Stata version V.16.0 (StataCorp). First, prevalence estimates stratified by sociodemographic variables (e.g., household income) were computed for all relevant child and maternal diet quality, for example, MDD, MDD‐W, MMF, MAD, and food insecurity indicators, that is, FIES at the household level, using data collected during COVID‐19/endline surveys.

Baseline prevalence estimates were extracted from reports and differences between baseline and endline data were calculated and expressed in percentage points.

### 2.5 Ethics statement

Ethical approval for all SDFU surveys was obtained from all the relevant authorities prior to data collection. The Government of Indonesia granted the research permit (Ministry of Internal Affairs Letter No. 460.02/012/DV) while ethical approvals for SDFUI2020 were granted by the University of Atma Jaya Ethical Committee (No. 0197/III/LPPM‐PM.10.05/02/2020 (for the previously planned face‐to‐face interview) and No. 0864/III/LPPM‐PM.10.05/08/2020 (for data collection method amendment to remote‐phone survey). Ethical approval for SDFUM 2018 was granted by the Myanmar Ethical Review Board (DMR) in 2018. It was not possible to seek separate approval for the SDFUM2021 follow‐up survey due to the political unrest in 2021, however, the same data collection protocols were followed as with SDFUM2018. In the Philippines, ethical clearance for SDFUP2020 was sought from a registered Ethical Review Board (ERB). Informed consent was obtained from participants before engagement for all study sites. Research teams collecting data respected the fundamental principles regarding research on human subjects and followed rigorous policies on data security and confidentiality as per FAO, WFP and UNICEF Data Management and Open Access Policy.

## 3 RESULTS

### 3.1 Characteristics of study participants

There were more households with CU5 surveyed in Jakarta than there were in Quezon City or Yangon. Of the three cities, Yangon had the largest proportion of impoverished households, with nearly 9 in 10 households living in extreme poverty (below the international poverty line of $1.90 per capita/day) (Table 1). Furthermore, there were fewer households classified as upper‐middle and nonpoor in Yangon, than there were in Jakarta.

**Table 1:** Sociodemographic characteristics of households and participants in Jakarta, Quezon City and Yangon.

Variable	Jakarta	Quezon City	Yangon
Households with children U5 (*n*)	777	2474	2201
*Children by age group (n, %)*:			
0–5 months	73 (9.40)	118 (4.33)	369 (16.77)
6–23 months	258 (33.2)	1248 (45.80)	845 (38.39)
12–23 months	158 (20.33)	795 (29.17)	527 (23.94)
24–59 months	446 (57.4)	1119 (41.06)	987 (44.84)
*Women 15–49 years with children (n, %)*:			
0–11 months	n/a	639 (23.44)	676 (30.71)
12–23 months	158 (20.33)	912 (33.47)	515 (23.40)
24–59 months	619 (79.66)	1174 (43.08)	1010 (45.89)
*Household income level (%)*:			
Extreme poverty	54.8	64.8	89.5
Lower middle‐income	29.6	35.2^a^	6.6
Upper middle‐income	10.4	^–^	1.2
Nonpoor (above all poverty lines)	5.1	^–^	6.2
*Mother's education level (n, %)*			
None or elementary	110 (14.16)	178 (6.53)	118 (5.36)
Primary	198 (25.48)	1306 (47.93)	826 (37.53)
Secondary	374 (48.13)	123 (4.51)	1080 (49.07)
Higher	95 (12.22)	812 (29.80)	177 (8.04)

^a^ Percent of all households above the international poverty line of $1.90 per capita/day.

**Figure 1:**
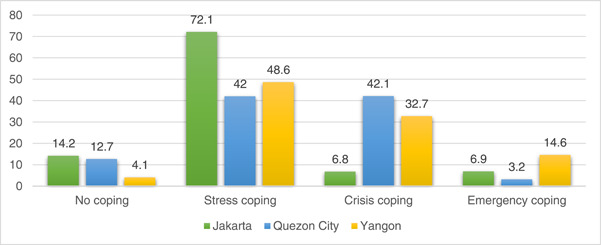
Coping strategies in response to changing household incomes in Jakarta (*n* = 777), Quezon City (*n* = 2474) and Yangon (*n* = 2201).

At least 80% of households in Jakarta and Quezon City, and up to 95% of households in Yangon reported a reduction in income with lower‐income households more severely affected by pandemic‐related income losses. In response, more than 8 in 10 surveyed households in all three cities, and more than 9 in 10 households in Yangon alone, applied at least one coping strategy (Figure 1), with the poorest households more likely to employ crisis and emergency coping strategies. Jakarta reported fewer households under the international poverty line (Table 1) and households reported employing less stringent coping strategies (no coping and stress coping), compared with households in Yangon, where nearly 90% of households were below the international poverty line (Table 1), and reported mostly resorting to crisis coping, for example, taking informal debt with interest, and emergency coping strategies, for example, begging and selling land or house (Figure 1).

### 3.2 Household food security and food purchasing patterns

**Figure 2:**
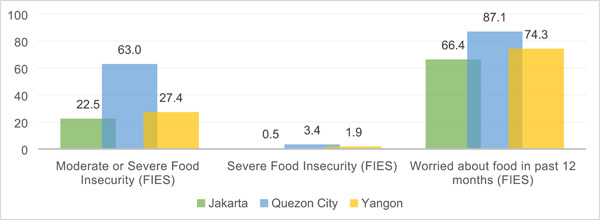
Food insecurity experience among surveyed households in Jakarta (*n* = 777), Quezon City (*n* = 2474) and Yangon (*n* = 2201).

At least 6 in 10 surveyed households in all three cities reported being worried about food in the year preceding the survey (Figure 2). Furthermore, findings indicate that at least 2 in 10 households reported experiencing moderate to severe food insecurity in all three cities (Figure 2). Quezon City appeared the most food insecure of all three cities following the pandemic, as it reported the highest proportion of participants both experiencing moderate or severe food insecurity (63% and 3.4, respectively) and being worried about food in the past 12 months (87.1%) (Figure 2).

In response to reduced incomes, a high proportion of surveyed households in Jakarta reported reducing their purchasing of nutrient‐dense foods, that is, meat, poultry and fish (61.6%), eggs (46.8%), fruit (49.4%), vegetables (28.6%) and beans, pulses and tofu (30.2%). In Yangon, households reported changing their purchasing of nutrient‐dense plant‐based foods, that is, beans, pulses, lentils, chickpeas, tofu (44.1%) and vegetables (39.4%), as well as animal‐based foods, that is, meat, poultry, fish, and shrimp (75.2%), eggs (72.8%). Here, at least 4 in 10 surveyed households who changed their food‐purchasing patterns bought smaller quantities of the aforementioned nutrient‐dense food groups, while at least 1 in 10 households substituted them for cheaper, less nutritious foods. Similar food‐based coping strategies were reported in Quezon City, where varying proportions of households refrained from purchasing corn (33%), meat (16%), organ meats (23%) and seafood (40%). Instead, households in Quezon City purchased cheaper substitutes, particularly when it came to breast milk substitutes, milk, and fish. While financial access played a role in changing food purchasing patterns in Yangon, findings indicated that reduced financial access was not significantly associated with changes in purchasing of pulses, meat, eggs, and vegetables in the city. Additionally, reduced physical access was not statistically significantly associated with changes in meat, egg and vegetable purchasing behaviour in Yangon or either of the other cities.

### 3.3 Impact of COVID‐19 pandemic on diet quality indicators among children under 5 years

MDD among children 6–23 months was lower by at least 15 percentage points (pp) from baseline, across all three cities, while MAD was the least achieved indicator among children 6–23 months. Findings indicated that it was harder for younger children (6–23 months) to achieve MDD compared with older ones (24–59 months), among whom higher proportions achieved this indicator in all the three urban‐poor settings (Table 2). Among children 6–23 months in Yangon and Jakarta, there was a lower intake of sugar‐sweetened beverages compared with prepandemic numbers (Table 2), although in Jakarta, there was also a reported decline in unhealthy food intake. Among older children (24–59 months), >5 in 10 reported eating any unhealthy food, with the proportion as high as 9 in 10 children in Jakarta (Table 2), although for children 25–59 months, little comparison could be made to prepandemic numbers for lack of data. Furthermore, more children 6–23 months in Quezon City than in Jakarta and Yangon did not consume any fruits and vegetables, with similar trends seen among older children (Table 2).

**Table 2:** Diet quality indicators among surveyed women and children 6–59 months in Jakarta, Quezon City and Yangon (compared with baseline where data are available).

Variable	Jakarta		Quezon City		Yangon	
	BL 2018	SDFUI 2020	BL 2013/2017	SDFUP 2020	BL 2018	SDFUM 2021
*Infant and young child feeding (6–23 months)* a						
Children fed minimum dietary diversity (MDD)	80.5	38.0	25.1	7.7	36.1	18.7
Children fed minimum meal frequency (MMF)	94.2	78.5	82.2	70.0	–	57.0
Children fed minimum acceptable diet (MAD)	75.8	31.6	14.6	6.6	–	12.0
Children fed any commercially fortified baby food	9.6	14.6	–	9.6	–	–
Children fed zero vegetable or fruit	9.6	12.7	–	69.0	–	57.9
Children fed unhealthy food	93.3	86.7	–	46.2	–	–
Children fed a sugar‐sweetened beverage	53.1	40.5	–	5.5	6.7	5.2
Children fed savoury and fried snacks	62.7	41.1	–	1.6	–	13
Food prepared for the child the previous day (12–23 months)	86.9	73.4	–	–	–	–
*Infant and young child feeding (24–59 months)* a						
Children fed minimum dietary diversity (MDD)	–	65.5	–	12.6	–	42.9
Children fed egg or any flesh foods	–	–	–	–	–	86.3
Children fed zero vegetable or fruit	–	21.5	–	57.2	–	–
Children fed unhealthy food	–	92.4	–	52.3	–	62.1
Children fed sugar‐sweetened beverage	–	–	–	13.5	–	–
Children fed savoury and fried snacks	–	–	–	6.5	–	41.0
*Women (including pregnant and lactating women)* a						
Women meeting minimum dietary diversity (MDD‐W)	78.9	74.1	–	15.7	61.5	34.0
Women consumed meat	89.8	72.2	–	89.6	–	–
Women consumed unhealthy food	90.9	80.4	–	15.1	–	–
Women consumed sugar‐sweetened beverage	83.6	65.2	–	46.6	51.3	–
Women consumed zero fruit or vegetables	8.3	7.6	–	39.9	–	–

Abbreviations: BL, baseline surveys; SDFU, SDFU surveys.

^a^ Based on food intake 24 h before survey.

### 3.4 Impact of COVID‐19 pandemic on diet quality indicators among women

Before the pandemic, at least 50% of women in Jakarta and Yangon met the MDD‐W, however, following the pandemic, this was lower in both cities, more so in Yangon where it reduced to only 34% (Table 2). In Quezon City, on the other hand, less than one in four women met the MDD‐W following the pandemic, but a lack of data made it incomparable with prepandemic levels. Findings indicated a small improvement in fruit and vegetable intake in Jakarta following the pandemic with only 7.6% of women reporting zero consumption in the 24 h before the survey (down from 8.3% at baseline) (Table 2). Jakarta saw a reduction in the proportion of women that consumed sugar‐sweetened beverages, with the figure dropping by about 20pp from baseline values to 65.2% (Table 2). Additionally, unhealthy food consumption among women in Jakarta fell by >10pp to 80.4% (Table 2). In Quezon City, only 15% of women reported eating unhealthy food the day or night before the survey (Table 2), but no prepandemic figures were available for comparison.

## 4 DISCUSSION

The SDFU surveys aimed to assess the impact of the COVID‐19 pandemic on food security and diet quality among women and children residing in urban poor areas of Jakarta, Quezon City and Yangon by comparing the change in selected indicators before and during the pandemic.

### 4.1 Food insecurity and food purchasing behaviour among urban‐poor households in Jakarta, Quezon City and Yangon

At least 20% of households in all three cities were moderately or severely food insecure, although Quezon City had the highest proportion of both moderately or severely food insecure households and households worried about food over the past year. Similar to our findings, an increase in pandemic‐related household food insecurity has been documented elsewhere (Chege et al., 2020; Kansiime et al., 2021; Madzorera et al., 2021; Matsungo & Chopera, 2020). Moreover, in line with our findings, IFPRI's Rural and Urban Food Security Survey (RUFSS)'s phone panel survey reported that 17% more households in Yangon reported reduced food consumption in the pandemic period 2020–2021 (International Food Policy Research Institute, 2021). These food consumption changes in Yangon in ours and IFPRI's survey could have resulted partly due to the compounding effect of a military coup in February 2021 and concurrent agricultural sector disruptions experienced as raw material procurement challenges, impaired transportation, and reduction in primary food production and processing (International Food Policy Research Institute, 2021). Similar disruptions significantly impacted food security in many other low, middle and high‐income countries, for example, the United Kingdom (Power et al., 2020; Shanks et al., 2020) and India (Harris et al., 2020), particularly in the earlier months of the pandemic, when lockdown measures were first implemented. However, while impactful, Fan et al. (2021) argue that these sector‐wide disruptions were unlikely to have equally impacted some Asian countries. Here, the authors reported that good transportation systems, robust food supply chains, reliable internet, widespread mobile phone use, and a culture of collective action for social good, buffered many ASEAN countries, including Indonesia, Myanmar, and the Philippines (Fan et al., 2021). Notwithstanding, it is pertinent to stress that while urban poor residents, such as those in our study, may reside within a ‘nationally resilient’ food system, their food security may still be compromised by other factors, for example, food accessibility and affordability. Moreover, urban areas, such as those included in our surveys, typically have longer and more complex food supply chains than rural areas, where people largely rely on own or local food production. Consequently, urban populations are typically more susceptible to even the smallest food system bottlenecks resulting from internal and external shocks, for example, restaurants and open food market closures (Akseer et al., 2020).

Various authors propose that pandemic‐related food insecurity could also have resulted from reduced working hours, unemployment, and delays or declines in remittances (Akseer et al., 2020; Amare et al., 2021; Diao & Mahrt, 2020; World Bank, 2020b; Zar et al., 2020), which could have collectively contributed to the reduced incomes seen in over 80% of surveyed households in our study. In the Philippines, for example, Murakami et al. (2020) projected that remittances would decline by 23%–32% through the first pandemic Year 2020, which would result in a 2.2%–3.3% decline in per capita household consumption. Findings from the aforementioned authors could account for findings from our study, where decreases in household incomes, due to restricted movement and unemployment, reportedly resulted in increased food insecurity (with the highest levels seen in the Philippines) and the use of food‐based coping strategies, that is, substituting more expensive ND foods, for example, meat, poultry, fruit and vegetables, with nutrient‐poor, cheaper alternatives, as well as buying smaller quantities of ND foods. Economic stress from movement restrictions was exacerbated by the fact that many urban‐poor residents are informally employed, earning low and unreliable incomes, which perpetuates urban food insecurity as this results in limited economic access to ND diets (Tacoli, 2019; Akseer et al., 2020; Vilar‐Compte et al., 2021). Already before the pandemic, a survey by the WFP and partners established that while 90% of households in Myanmar could afford a diet sufficient to meet their energy needs, only 40% could afford a healthier diet (WFP World Food Programme, 2019). Declines in income would have made such ND diets even more unattainable for many households in Yangon. Moreover, economic challenges in Yangon would have been worsened by the concurrent political crisis.

### 4.2 Diet quality among children under 5 years and women in urban‐poor households in Jakarta, Quezon City and Yangon

MDD among children (6–23 months) was lower by >15pp from baseline, across all three cities. In their papers, Akseer et al. (2020) and Zar et al. (2020) reported diet quality among CU5 in LMIC being indirectly affected by in‐access to school feeding and disruptions in regular child nutrition services, for example, supplementation. While we did not collect any data on school attendance among CU5, given the widespread restrictions in movement enforced in all three cities, we can postulate that these observations could partly explain some of our findings regarding declines in diet quality among CU5, in addition to the previously highlighted factors that impacted overall household food security. On the other hand, intake of energy‐dense, nutrient‐poor foods generally dropped among both CU2 and CU5, although both age groups still consumed sugar‐sweetened beverages and sweet and savoury snacks. These changes could have directly resulted from a loss in disposable income, as well as movement restrictions so that shops selling these items were no longer accessible. Furthermore, a recent study on food consumption patterns among urban‐poor and rural families in the Philippines found that, for convenience among other factors, urban families often opted for cheaper processed foods, which consequently shaped their children's dietary preferences towards choosing high sugar and high‐fat foods and vegetable avoidance (Siy Van et al., 2021). This was similar to Akseer et al., 2020 paper, where the authors reported a substitution of nutrient‐dense foods with cheaper options as a common coping strategy in many LMIC during the pandemic. This means that despite changes in pandemic‐related physical and economic access, CU5 were still likely to consume energy‐dense, nutrient‐poor foods.

In Quezon City, less than 25% of women met the MDD‐W, while the proportion of women meeting the MDD‐W in Jakarta and Yangon dropped from baseline figures. While few studies have reported on the impacts of the pandemic on MDD‐W in LMIC (Picchioni et al., 2022), based on other indicators, a decline in overall diet quality was reported among women in South Africa (Nyashanu, Simbanegavi, et al., 2020), Kenya and Uganda (Kansiime et al., 2021), Zambia (Nyashanu, Deborah, et al., 2020) and Mexico (Batis et al., 2021). Similar to CU5, although some declines in energy‐dense, nutrient‐poor food intake were observed, there was still intake of these foods during the pandemic survey period. In their modelling study, Laborde et al. (2021) projected that, during the pandemic, households in 63 low and middle‐income countries would substitute ND foods with increased intakes of cheaper vegetable oils, sugar, processed foods and food away from home, as a coping strategy. Furthermore, in addition to financial constraints, a study among Mexican women, found that decreases in unhealthy food intake at the start of the pandemic, and then increases later on in the pandemic, were linked with many factors, including stockpiling of junk food, increased need for comfort eating, and reduced interest in healthy eating (Batis et al., 2021). Some of these factors could likely explain why women in our study still consumed sugar‐sweetened beverages and sweet and savoury foods during the pandemic. Furthermore, restrictions could have directly compromised diet quality as many women could have had reduced access food to outside the home (Laborde et al., 2021), some of which could still be healthy, for example, ready‐cooked plant‐based meals.

### 4.3 Strengths and limitations

This study explored the effects of the pandemic on food security and diet quality among both children and women in urban‐poor settings in three diverse Asian countries using the food security experience scale, increasing the likelihood of robust and comparable data. These findings provide valuable information on food security among the urban poor in these cities and are suggestive of what might be prioritised in the face of similar pandemics in the future. Nevertheless, both baseline and SDFU surveys were cross‐sectional in nature, making it difficult to establish causality between outcome and independent variables and to extrapolate results to the wider urban‐poor population. However, while the results are not representative of all urban areas within the respective countries, they provide credible data on the situation among the surveyed urban‐poor communities. Lastly, the results on the impact of COVID‐19 and response measures dynamically changed, meaning that the findings from these surveys are only representative of data collected during a specific time. Timely monitoring is needed to provide regular data on food security and diet quality as countries recover from the pandemic.

## 5 CONCLUSION

Food insecurity and diet quality among urban‐poor children and mothers in all three cities worsened following the pandemic, partly due to reduced incomes, which resulted in higher household poverty, more so among households in Yangon. Although food insecurity was high across all three cities, the highest figures were seen in Quezon City. There was a decline in the proportion of children 6–23 months and mothers achieving the MDD across all three cities. Energy‐dense, nutrient‐poor food intakes were still reported among mothers and children in all cities, although the proportion was lower compared with baseline. To reduce food insecurity and improve diet quality, multi‐sectoral initiatives are needed to: (1) use robust data management systems to streamline comprehensive eligibility criteria for food and cash aid to cover all vulnerable groups, for example, CU2, adolescent girls, and extremely poor households (Akseer et al., 2020); (2) during stress periods, for example, pandemics, promote distribution of ND food baskets containing fruit, vegetables and flesh foods through large government‐led social protection programmes, among nutritionally vulnerable households; (3) concurrently with food baskets, provide culturally‐appropriate complementary training to improve knowledge of healthy dietary behaviours and optimal IYCF practices to facilitate the increase in household expenditure of cash benefits on ND foods to improve childhood stunting (Conway et al., 2020; Huicho et al., 2020; Tasic et al., 2020); (4) in the absence of physical infrastructure for complementary feeding training and nutrition services, use mobile clinics, community‐based health workers, and local women's groups, which have been successful in the delivery of health‐related services in other resource‐poor contexts; and (5) detect and quickly manage those vulnerable to malnutrition in the community (Ntambara & Chu, 2021).

## AUTHOR CONTRIBUTIONS

Carolyn I. Auma and Sophie Goudet developed the concept for the paper. Kristian A. Brizendine, Sabrina Eymard‐Duvernay and Sophie Goudet extracted and analysed primary data, Carolyn I. Auma, and Sophie Goudet, critically analysed and synthesised the literature. Carolyn I. Auma wrote the first draft of the manuscript with critical input from Sophie Goudet, Rebecca Pradeilles, Heather Ohly, Jessica Blankenship, and Sabrina Eymard‐Duvernay. All authors read and approved the final version of the manuscript.

## CONFLICT OF INTEREST STATEMENT

The authors declare no conflict of interest.

## Supporting information

Supporting information.

Supporting information.

## Data Availability

The data that support the findings of this study are available from the corresponding author upon reasonable request.
