# Supplementary material for: Urban nutrition situation in the slums of three cities in Asia during the COVID‐19 pandemic
Source: Matern Child Nutr. 2023 Oct 9;22(1):e13543. doi: 10.1111/mcn.13543 (PMC12647976; doi:10.1111/mcn.13543)
Supplement: Supplementary file 1 — Supporting information. [file MCN-22-e13543-s002.docx]

**Supplementary Material 1**

**Sampling frame, study sites and questionnaires for SDFU surveys**

1. Jakarta

Table 1: List of locations for data collection

| Kota | Subdistrict | Village | Hamlet |
| --- | --- | --- | --- |
| Jakarta Barat | Grogol Petamburan | Wijaya Kusuma | RW 2 - Wijaya Kusuma |
|  | Kebon Jeruk | Duri Kepa | RW 2 - Duri Kepa |
|  | Pamerah | Jati Pulo | RW 7 - Jati Pulo |
|  |  |  | RW 8 - Jati Pulo |
|  |  |  | RW 9 - Jati Pulo |
|  | Taman Sari | Krukut | RW 3 - Krukut |
|  | Grogol Petamburan | Tomang | RW 11 - Tomang |
|  |  |  | RW 14 - Tomang |
|  | Kali Deres | Semanan | RW 10 - Semanan |
|  |  | Tegal Alur | RW 8 - Tegal Alur |
|  | Pamerah | Palmerah | RW 4 - Palmerah |
|  | Taman Sari | Keagungan | RW 2 - Keagungan |
|  | Tambora | Jembatan Besi | RW 2 - Jembatan Besi |
|  |  |  | RW 7 - Jembatan Besi |
|  |  | Pekojan | RW 10 - Pekojan |
| Jakarta Timur | Cakung | Cakung Barat | RW 7 - Cakung Barat |
|  | Jatinegara | Kampung Melayu | RW 2 - Kampung Melayu |
|  |  |  | RW 3 - Kampung Melayu |
|  |  |  | RW 8 - Kampung Melayu |
|  | Kramat Jati | Kampung Tengah | RW 7 - Kampung Tengah |
|  | Makasar | Pinang Ranti | RW 2 - Pinang Ranti |
|  | Pasar Rebo | Pekayon | RW 4 - Pekayon |
|  | Pulogadung | Kayu Putih | RW 15 - Kayu Putih |
|  | Jatinegara | Cipinang Besar Selatan | RW 2 - Cipinang Besar Selatan |
|  |  |  | RW 9 - Cipinang Besar Selatan |
|  |  | Cipinang Besar Utara | RW 7 - Cipinang Besar Utara |
|  |  | Rawa Bunga | RW 1 - Rawa Bunga |
|  |  |  | RW 5 - Rawa Bunga |
|  | Makasar | Cipinang Melayu | RW 3 - Cipinang Melayu |
| Jakarta Utara | Kelapa Gading | Pegangsaan Dua | RW 2 - Pegangsaan Dua |
|  |  |  | RW 3 - Pegangsaan Dua |
|  | Koja | Tugu Selatan | RW 4 - Tugu Selatan |
|  | Penjaringan | Kamal Muara | RW 4 - Kamal Muara |
|  | Tanjung Priok | Papanggo | RW 5 - Papanggo |
|  |  | Sunter Agung | RW 1 - Sunter Agung |
|  |  | Sunter Jaya | RW 2 - Sunter Jaya |
|  |  | Tanjung Priok. | RW 7 - Tanjung Priok |
|  |  |  | RW 15 - Tanjung Priok |
|  | Cilincing | Semper Barat | RW 16 - Semper Barat |
|  | Pademangan | Ancol | RW 4 - Ancol |
|  |  | Pademangan Timur | RW 10 - Pademangan Timur |
|  | Penjaringan | Penjaringan. | RW 4 - Penjaringan |
|  |  |  | RW 11 - Penjaringan |
|  |  |  | RW 14 - Penjaringan |
|  |  |  | RW 15 - Penjaringan |
|  |  |  | RW 16 - Penjaringan |

Table 2: Sample size estimates

| Indicator | Population | Estimates (%) | Margin of error (%) | Design effect | Sample size |
| --- | --- | --- | --- | --- | --- |
| Estimates from REC endline (2019) | | | | | |
| Minimum diet diversity | Children 12-23 months | 72.9 | 10 | 2 | 152 |
| Minimum acceptable diet |  | 63.6 | 10 | 2 | 178 |
| Minimum meal frequency |  | 94.2 | 5 | 2 | 168 |
| Minimum dietary diversity for women | Mother of children 12-23 months | 78.9 | 10 | 2 | 128 |
| Food insecurity: all | Household of children under 2 years | 59.7 | 10 | 2 | 185 |
| Food insecurity: mild |  | 23.5 | 10 | 2 | 138 |
| Food insecurity: moderate |  | 17.2 | 10 | 2 | 109 |
| Food insecurity: severe |  | 18.9 | 10 | 2 | 118 |
| Estimates from Urban in Bandung | | | | | |
| Minimum diet diversity | Children 6-23 months, urban low SES | 73.7 | 10 | 2 | 149 |
| Minimum acceptable diet | Children 6-23 months, urban low SES | 57.3 | 10 | 2 | 188 |
| Minimum meal frequency | Children 6-23 months, urban low SES | 76.7 | 10 | 2 | 137 |

1. Quezon City

Table 1: List of locations for data collection

| District | Villages |
| --- | --- |
| 1 | Alicia  Bahay Toro  Santa Domingo |
| 2 | Bagong Silangan  Commonwealth  Payatas |
| 3 | Botocan  Matandang Balara  Pansol |
| 4 | Krus na Ligas  Obrero  San Isidro  San Martin De Porres  Tatalon |
| 5 | Capri |
| 6 | Baesa  Balintawak |

For sample size calculations, the formula used was:

n = required minimum sample size per comparison group

p = the estimated level of an indicator measured as a proportion for the control area

Z = the Z-score corresponding to the degree of confidence (1.96 for 95% confidence)

c = confidence interval

Table 2: Parameters and associated values and assumptions for sample size estimates

|  | Parameter | Value | Assumption |
| --- | --- | --- | --- |
| p | the estimated level of an indicator measured as a proportion for the control area | 44.20% | HFA expected based on urban studies (NNS lowest health quintile 2013) |
| z@95% | the Z-score corresponding to the degree of confidence | 1.645 | 95% |
|  | Target group as % of the population | 13.50% | Estimated based Philippines census 2015 |
|  | % of non-response | 20% | Estimated based on response rate for 2018 ENNS (households) |
|  | Average HH size | 3 | Estimated based Philippines census 2015 |
|  | Number of HH required | 1. 000 |  |

1. Yangon

Table 1. Sample points and HH per township used in SDFUM 2018

|  | Formal settlements | | Informal settlements | |
| --- | --- | --- | --- | --- |
| Township | **Total Population included** | **Sample HHs** | **Total Population included** | **Sample HHs** |
| Dagon Myothit (East) | 156,244 | 80 | 15,100 | 80 |
| Dagon Myothit (Seikkan) | 167,448 | 90 | 48,489 | 90 |
| Dagon Myothit (South) | 371,646 | 190 | 8,395 | 190 |
| Dala | 119,366 | 60 | 29,920 | 60 |
| Hlaingtharya | 687,867 | 360 | 86,046 | 360 |
| Insein | 305,283 | 160 | 53,935 | 160 |
| Kyeemyindaing | 22,303 | 20 | 20,250 | 20 |
| Mingaladon | 331,586 | 170 | 18,645 | 170 |
| North Okkalapa | 333,293 | 170 | 29,185 | 170 |
| Seikgyikanaungto | 34,003 | 20 | 24,521 | 20 |
| Shwepyithar | 343,526 | 180 | 10,040 | 180 |
|  | **2,872,565** | **1,500** | **344,526** | **1,500** |

Table 2. Sample size calculation^[[1]](#footnote-2)^

**Data Collection Questionnaires**

**Urban study research REMOTE “Status and determinants of food insecurity and undernutrition in poor urban areas” during C19 (SDFU-2020)**

**HOUSEHOLD SURVEY QUESTIONNAIRE (HHQ)**

**(THE QUESTIONNAIRE WILL BE ADMINISTRATED BY PHONE)**

**INFORMED CONSENT (3Q) PAGE 1**

**SECTION 1 – HOUSEHOLD PROFILE (5Q) PAGE 1**

**SECTION 2 - EMPLOYMENT SITUATION AND CONDITIONS, REMITTANCE RECEIVED (7Q) PAGE 2**

**SECTION 3 – CREDITS/DEBTS AND REMITTANCES (2Q) PAGE 4**

**SECTION 4 – HOUSING (5Q) PAGE 4**

**SECTION 5 – CHANGE IN MARKET FOOD ACCESS IN C19 CONTEXT (1Q-11 ITEMS) PAGE 5**

**SECTION 6 – FOOD INSECURITY EXPERIENCE SCALE (8Q) PAGE 6**

**SECTION 7 – WATER, SANITATION, AND HYGIENE (WASH) (4Q) PAGE 7**

**SECTION 8 – COVID 19 SHOCK (1Q -14 ITEMS) PAGE 8**

**SECTION 9 – FOLLOW UP (3Q) PAGE 8**

**Date**|___||___|/|___||___|/|_2_||_0_| **(DD/MM/YY) TIME STARTED: |__||__|:|__||__| TIME ENDED: |****__||__|:|__||__|**

**Introduction**: Hello Ma’am, pwede po kay <name of respondent>? Hi Ma’am <name of respondent>, ako po si <name of enumerator> at ako po ay tumatawag bilang isang enumerator ng UP Los Baños, FAO, at iba pang UN Agencies. Sa kasalukuyan, kami po ay nagsasagawa ng pag-aaral upang lalo pang maintindihan ang seguridad ng pagkain, nutrisyon, kalusugan, at kalinisan ng inyong tahanan. Noong huling tawag po namin sa inyo, nasabi ninyo po na kayo ay pumapayag na lumahok sa aming pag aaral. Tama po ba Ma’am <name of respondent>? <wait for answer> Okay po, Ma’am.

Ma’am ang panayam po ay magtatagal ng higit kumulang 15 minuto. Maari po bang mainterview ko na po kayo ngayon? <wait for answer> Okay po, maraming salamat po <if no, ask for a better time to do the interview>. Ma’am, kung sakali pong maputol ang aking tawag sa inyo, ay agad naman po akong tatawag muli para ipag-patuloy ang ating panayam. Lahat ng inyong impormasyon ay aming ituturing na pribado at gagamitin lamang sa layunin ng pag-aaral na ito. Para sa aming pasasalamat sa inyong paglahok, magpapadala kami ng [AMOUNT] load sa numero na ito. Pwede na po ba natin simulan ang panayam? <Wait for answer>

| **Informed consent (3 QUESTIONS)** | |  |  |
| --- | --- | --- | --- |
| IC.1 | Do you agree to participate in this study?  *Sumang-ayon po ba kayo na makilahok sa pagaaral na ito?* | 1. Yes *(Oo)* 2. No *(Hindi)*, bakit? __________________ | \|___\| |
| IC.2 | Are you a mother of U5 children?  *Kayo po ba ay ina ng batang may edad na limang taon o mababa sa limang taon?*  [IF YES, MENTION THIS STATEMENT TO THE PARTICIPANT “BALI MA’AM, MAGTATANONG DIN PO AKO NG MGA PATUNGKOL SAINYO AT SA PINAKABATA NINYONG ANAK”] | 1. Yes *(Oo)* => **use** **MCQ at the end of HHQ** 2. No *(Hindi)* => **Proceed with IC.3** | \|___\| |
| IC.3 | Is there a mother of U5 in your HH?  *Mayroon po bang ina ng batang may edad na limang taon o mababa sa limang taon sa inyong bahay?*  [IF YES, MENTION THIS: “MA’AM MAAARI NYO PO BANG MAIPASA ANG TELEPONO SAKANYA **MAMAYA (DURING MCQ)** UPANG SIYA ANG AMING TANUNGIN PATUNGKOL SA PINAKA BATANG ANAK? MAAARI DIN PO NA MAGBIGAY KAYO NG KANYANG NUMERO UPANG SIYA AY DIREKTA NAMING MATAWAGAN”] | 1. Yes *(Oo)* => **use** **MCQ at the end of HHQ** 2. None *(Wala)* => **continue with HHQ only**  **If yes, use MCQ at the end of HHQ**  **If yes, get the cellphone number**  **________________________________** | \|___\| |
| **Section 1 – HOUSEHOLD PROFILE (5 QUESTIONS)** | | | |

| 1.1 | What is the number of persons living in your household?  *Ilan po kayong nakatira sa inyong bahay?*  A household is defined as a group of people **currently** eating from the same pot “under the same roof” (or in same compound if the HH has 2 structures). *Ang isang sambahayan ay grupo ng mga tao na magkakapamilya o hindi na kasalukuyang kumakain sa iisang hapag kainan.* | | | | | | | | | | \|___\|\|___\| | |  |  |
| --- | --- | --- | --- | --- | --- | --- | --- | --- | --- | --- | --- | --- | --- | --- |
| 1.2 | How many children under 5 are living in your household?  *Ilan po ang batang may edad na limang taon o mababa sa limang taon?* | | | | | | | | | | \|___\|\|___\| | |  |  |
| 1.3 | Are you originally staying/living in that area?  *Palagi po ba kayong nakatira o orihinal po ba kayong nakatira diyan sa inyong lugar.* | | | | 0=Always lived here *(dito laging nakatira)* => **Proceed to 1.5**  1=Did not always live here *(hindi dito lagi nakatira)*  2=Used to live here, left and recently moved back here *(nakatira dito dati, umalis at bumalik ulit ngayon*  98=Do not know *(hindi alam)*  Others, specify: _____________________________ | | | | | | \|___\|\|___\| | |  |  |
| 1.4 | Did you move to this location because of the COVID19 epidemic (from the period of March-July 2020) from a different city or village?  *Kayo po ba ay lumipat lamang diyan mula sa ibang siyudad o barangay dahil sa COVID19 pandemic (mula Marso-Hulyo 2020)?* | | | | 0=No *(Hindi)*  1=Yes *(Oo)* | | | | | | \|___\| | |  |  |
| 1.5 | What is the gender of the household head?  *Ano po ang kasarian ng pinuno ng inyong tahanan?* | | | | 1=Male *(Lalaki)*  2=Female *(Babae)* | | | | | | \|___\| | |  |  |
| **SECTION 2. Employment situation and conditions, remittance received (7 QUESTIONS)** | | | | | | | | | | | | |  |  |
| 2.1 | | How many are earning in your family?  *Ilan po ang kumikita sa inyong pamilya?* | | | | | | \|___\|\|___\| | | | | |  |  |
| 2.2 | | What are the sources of income of your household?  *Ano-ano po ang mga pinag-kakakitaan ng inyong pamilya?*  [DO NOT READ THE CHOICES. LET THE RESPONDENT ANSWER] | | | | | | 1 = Job/Work  2 = Own business (sari-sari store, carinderia, seller, etc.)  3 = Farmer/Fisherfolk  4 = Pension  5 = OFW/remittances  6 = Others, specify ______________________________ | | | \|___\|  \|___\|  \|___\|  \|___\| | |  |  |
| 2.3 | | How much is the usual gross income of each working family member every month, in Philippine peso?  *Magkano po ang karaniwang kinikita (gross) ng bawat miyembro ng inyong pamilya na nagtatrabaho? (kada buwan, in Philippine peso)* | | | | | | 1^st^ miyembro: Php ______________________  ___ miyembro: Php ______________________  ___ miyembro: Php ______________________  ___ miyembro: Php ______________________  ___ miyembro: Php ______________________  TOTAL (kada buwan): PHP ____________________________ | | | | |  |  |
| 2.4 | | How would you compare your household income now to your income at the start of the year (January 2020)?)  *Paano po ninyo maikukumpara ang kinikita ng inyong tahanan ngayon sa kinikita ninyo noong simula ng taon (Enero 2020)?* | | | | | | 0=No change *(Walang pagbabago)* => **Proceed to 2.6**  1=Increased income *(Tumaas ang kita)*  2=Reduced income *(Bumaba ang kita)*  98=Do not know *(Hindi alam)* =>  **Proceed to 2.6** | | | | \|___\|\|___\| |  |  |
| 2.5 | | Currently, what are the causes of the change in your household income?  *Ano-ano ang mga dahilan sa pagbabago ng kinikita ng inyong tahanan ngayon?*  [DO NOT READ THE CHOICES. LET THE RESPONDENTS ANSWER. IF THE RESPONDENT DID NOT MENTION THE OPTION, INDICATE “0”]  0 = No *(Hindi)*  1 = Yes *(Oo)* | | | | | | 1= Could not work due to travel/movement restrictions *(Hindi makapagtrabaho dahil sa paghihigpit sa byahe at paglabas-labas)*  2=Disruptions in markets (not able to sell products or buy inputs) *(Pagka-antala sa merkado (hindi makapagbenta o makabili ng produkto)*  3= Less customers/clients *(Kaunting customer o mamimili)*  4= Had to close shop/business *(Kailangang isarado ang tindahan o negosyo)*  5= Household members working are sick or in quarantine  *(Nagsakit o naka-quarantine ang miyembro ng pamilya)*  6=Loss of employment *(Nawalan ng trabaho)*  7=Reduced salary/wage *(Nabawasan ang sweldo o kita)*  8=Daily labour opportunities reduced *(Nabawasan ang maaaring pagkakitaan)*  9=Support/assistance has been reduced *(Nabawasan ang tulong o suporta)*  10=Have a new job *(May bagong trabaho)*  11=Increasing number of customers/*clients (Tumaas ang dami ng customer o mamimili)*  12=The price of goods or services increases *(Tumaas ang presyo ng mga bilihin o mga serbisyo)*  13=Receive income assistance (from the government)  *(Nakatanggap ng tulong mula sa gobyerno)*  14=Other (specify)_________________________________ | | | | **\|___\|**  **\|___\|**  **\|___\|**  **\|___\|**  **\|___\|**  **\|___\|**  **\|___\|**  **\|___\|**  **\|___\|**  **\|___\|**  **\|___\|**  **\|___\|**  **\|___\|**  **\|___\|** |  |  |
| 2.6 | | Now I will ask questions on strategies that your household may or may not be using to cope with the current situation.  *Ngayon magtatanong naman po ako tungkol sa mga estratehiya na ginagamit ng inyong pamilya upang makayanan ang kasalukuyang sitwasyon.*  During the past 30 days, did anyone in your family have to engage in any of the following activities because there was not enough resources (food, cash, etc.) to access essential needs (e.g. adequate shelter, education services, health services or other basic needs)?  *Nitong nakaraang 30 araw, mayroon po bang miyembro ng inyong pamilya na kinailangang gawin ang alinman sa mga sumusunod dahil kulang ang pagkain o pera upang makabili ng mga pangunahing pangangailangan?*  [READ ALL THE ACTIVITIES TO THE RESPONDENT. IF THE ANSWER IS NO, ASK WHY. PLACE APPROPRIATE CODE TO EACH ACTIVITY BASED ON THE ANSWER OF THE RESPONDENT.]  1 = Yes *(Oo)*  2 = No, why? *(Hindi, bakit?) _______________*  3 = N/A, Specify why | | | | | | 1. Sold household assets/goods (radio, furniture, refrigerator, television, jewelry…) *(Nagbenta ng mga gamit sa bahay tulad ng radyo, mga kasangkapan, refrigerator, telebisyon, at mga alahas.)* | | | | \|___\| |  |  |
|  |  |  |  |  |  |  |  | 1. Reduced non-food expenses on health (including drugs) *(Nagbawas ng mga gastusin na may kinalaman sa kalusugan, kasama ang mga gamot)* | | | | \|___\| |  |  |
|  |  |  |  |  |  |  |  | 1. Sold productive assets or means of transport (sewing machine, wheelbarrow, bicycle, car…etc.) *(Nagbenta ng mga pinagkakakitaang ari-arian o mga gamit sa transportasyon tulad ng mga makinang pangtahi, bisikleta, at iba pa.)* | | | | \|___\| |  |  |
|  |  |  |  |  |  |  |  | 1. Spent savings *(Ginamit ang ipon)* | | | | \|___\| |  |  |
|  |  |  |  |  |  |  |  | 1. Borrowed money/food from a formal lender/bank *(Nangutang ng pera at/o pagkain mula sa mga pormal na institusyong pang-pinansiyal tulad ng bangko)* | | | | \|___\| |  |  |
|  |  |  |  |  |  |  |  | 1. Sold house or land *(Nagbenta ng bahay o lupa)* | | | | \|___\| |  |  |
|  |  |  |  |  |  |  |  | 1. Withdrew children from school *(Pinahinto ang mga anak sa pagpasok sa eskwela dahil sa kakulangan ng pera)* | | | | \|___\| |  |  |
|  |  |  |  |  |  |  |  | 1. Sold last female animals *(Ibinenta ang huling alagang inahing hayop)* | | | | \|___\| |  |  |
|  |  |  |  |  |  |  |  | 1. Sold more animals (non-productive) than usual *(Nagbenta ng mga alagang hayop na ang dami ay higit sa pangkaraniwang ibinebenta)* | | | | \|___\| |  |  |
|  |  |  |  |  |  |  |  | 1. Pawning (Nagsangla ng gamit) | | | | \|___\| |  |  |
|  |  |  |  |  |  |  |  | 1. Begging *(Namalimos)* | | | | \|___\| |  |  |
|  |  |  |  |  |  |  |  | 1. Others, specify _____________________________ | | | | \|___\| |  |  |
| 2.7 | | Starting January 2020, did you receive social protection program or support from the government or other private organizations?  *Simula noong Enero 2020, nakatanggap po ba kayo ng social protection program o tulong mula sa gobyerno o pribadong organisasyon?* | | | | | | 0=No *(Hindi)* => **Proceed to 3.1**  1= Yes *(Oo)* => **Proceed to 2.8** | | | | \|___\| |  |  |
| 2.8 | | What kind of social protection program or support have you received from the government or private organizations?  *Anong uri po ng* *social protection program o tulong mula sa gobyerno o pribadong organisasyon ang inyong natanggap?* | | | | | | 1 = Targeting cash assistance *(Ayudang pera)*  2 = Food subsidies *(Ayudang pagkain)*  3 = Others, specify _____________________________________ | | | | \|___\| |  |  |
| **SECTION 3. Credits/Debts and remittances (2 QUESTIONS)** | | | | | | | | | | | | |  |  |
| **3.1** | | In the last 30 days, did anyone in your household borrowed money?  *May miyembro po ba ng inyong sambahayan ang nagutang nitong nakaraang buwan?* | | | | | 0 = No *(Hindi)*  => **Proceed to Section 4.1**  1 = Yes *(Oo)* | | | | | \|___\| |  |  |
| **3.2** | | Where was the borrowed money used for?  *Saan po ginamit ang inyong inutang?*  [DO NOT READ THE CHOICES. LET THE RESPONDENTS ANSWER. IF THE RESPONDENT DID NOT MENTION THE OPTION, INDICATE “0”]  0 = No *(Hindi)*  1 = Yes *(Oo)* | | | | | 1. Food purchases (*Pambili ng pagkain)* | | | | | \|___\| |  |  |
|  |  |  |  |  |  |  | 1. Health expenditures *(Gastusin na pangkalusugan)* | | | | | \|___\| |  |  |
|  |  |  |  |  |  |  | 1. Water purchase (both domestic and drinking) *(Pambili ng tubig na inumin at tubig pang gamit sa bahay)* | | | | | \|___\| |  |  |
|  |  |  |  |  |  |  | 1. House maintenance/improvement *(Pagpapabuti at pagpapanatili ng bahay)* | | | | | \|___\| |  |  |
|  |  |  |  |  |  |  | 1. Sanitation facility construction / maintenance *(Pagpapabuti o pagpapagawa ng pasilidad pang palikuran)* | | | | | \|___\| |  |  |
|  |  |  |  |  |  |  | 1. House purchase *(Pambili ng bahay)* | | | | | \|___\| |  |  |
|  |  |  |  |  |  |  | 1. Education *(Pag-aaral)* | | | | | \|___\| |  |  |
|  |  |  |  |  |  |  | 1. Funeral / wedding *(Libing/Kasal)* | | | | | \|___\| |  |  |
|  |  |  |  |  |  |  | 1. Business investment *(Puhunan sa negosyo)* | | | | | \|___\| |  |  |
|  |  |  |  |  |  |  | 1. Other debts reimbursement *(Pang bayad sa ibang utang)* | | | | | \|___\| |  |  |
|  |  |  |  |  |  |  | 1. Migration costs (travel within country or abroad) *(Paglipat ng tirahan (sa loob o labas ng bansa)* | | | | | \|___\| |  |  |
|  |  |  |  |  |  |  | 1. Utility bills (water, electricity, phone, internet) *(Bayarin sa tubig, kuryente, telepono, internet)* | | | | | \|___\| |  |  |
|  |  |  |  |  |  |  | 1. Other, specify ___________________________ | | | | | \|___\| |  |  |
| **SECTION 4. Housing (5 QUESTIONS)** | | | | | | | | | | | | |  |  |
| **4.1** | | What is the name of your sitio?  *Ano po ang pangalan ng inyong sitio?* | | | |  | | | | | | |  |  |
| **4.2** | | What is your complete address?  *Ano po ang inyong buong address?* | | | |  | | | | | | |  |  |
| **4.3** | | How many rooms do you have in your house (including bedrooms, living room, work rooms)? Do not include restrooms, kitchen, hallway, etc.  *Gaano kadaming kwarto ang inyong bahay kabilang ang tulugan, sala, o kwarto pang negosyo? Hindi kasali sa bilang ang banyo, kusina, pasilyo, atbp* | | | | | | | | | | \|___\| |  |  |
| **4.4** | | Do you own your house and lot? [If not, ask the respondent if the house is rented, under mortgage, use without pay, or temporary squatting]  *Sarili po ba ninyo pag mamay-ari ang tinitirhan ninyong bahay at lupa? [Kung hindi, itanong kung ito ay inuupahan, hinuhulugan, libreng pinatitiram o pansamantalang pinatitira lamang]*  [READ THE CHOICES AND LET THE RESPONDENT ANSWER] | | | | | | | | 1=Rented *(Inuupahan)*  2=Owned *(Pag mamay ari)*  3=Mortgage *(Hinuhulugan)*  4=Use without pay *(Walang bayad/Libreng tinitirhan)*  5=Temporary squatting *(Tinitirhan pansamantala ng walang kaukulang permiso)*  6=Others, specify ___________________________ | | \|___\| |  |  |
| **4.5** | | What is the type of your house? Is it <site choices>?  *Ano po ang uri ng inyong tinitirhan? Ito ba ay <site choices>?*  [LET THE RESPONDENT DECRIBE FIRST THEIR HOUSE. SUGGEST/CITE THE CHOICES ONLY IF NEEDED.] | | | | | | | | 0=Stand-alone /Single-detached *(Solo na bahay)*  1=Hut *(Bahay kubo)*  2=Workers room *(Kwarto ng mang gagawa)*  3=Dormitory *(Dormitoryo)*  4=Flat *(Apartment)*  5=Made from different construction materials (Tagpi-tagping bahay)  6=Others, specify _____________________________ | | \|___\| |  |  |
| **SECTION 5. CHANGE IN HH MARKET ACCESS IN C19 CONTEXT (1 QUESTION – 11 ITEMS)** | | | | | | | | | | | | | |  |
| Did you change the way you buy the following food items (5.1-5.11) since the start of the lockdown due to Covid19 pandemic?  *Kayo po ba ay nagbago ng pamamaraan ng pagbili ng mga ng mga sumusunod na pagkain (5.1-5.11) simula noong paghihigpit at lockdown dahil sa Covid19 pandemic?*  [READ EACH FOOD GROUP THEN ASK IF THERE IS A CHANGE IN MANNER OF PURCHASING. IF YES, ASK WHAT HAS CHANGED. HOWEVER, DO NOT READ THE CHOICES FOR THE QUESTION “WHAT HAS CHANGED?” DO THE SAME FOR THE REST OF THE FOOD GROUPS.] | | | | **Pagbabago**  0 = No *(Hindi)*  1 = Yes *(Oo)*  2 = Not applicable/never bought *(Hindi bumibili kailanman)* | | | | | **If yes,** what has changed?  1=Having more of this food delivered *(Mas maraming klase ng pagkain na ito ang pina-deliver)*  2=Buying more of this food from restaurants or street vendors *(Mas maraming klase ng pagkain na ito ang binibili mula sa mga nag bebenta sa kalsada o sa restaurant)*  3= Buying more of this food from market or shops making fewer trips to go shopping *(Mas maraming klase ng pagkain na ito ang binibili sa palengke upang hindi pabablik-balik sa pamilihan)*  4= Substitute the usual food items with other cheaper food items *(Pinapalitan ang mga binibiling karaniwang klase ng pagkain sa mga mas mura na klase)*  5=Buying less of this food because reduced purchasing power/increasing prices *(Mas kaunti ang binibili na pagkain na ito dahil sa pagliit ng budget/pagtaas ng presyo)*  6= Buying less of this food because reduced access to functioning/open market facilities (physical access problem) *(Mas kaunti ang binibili na klase ng pagkain na ito dahil sa pag sasara ng ibang mga pamilihan )*  7=Stopped buying because not available in the market *(* *Hindi na bumibili ng klase ng pagkain na ito dahil walang mabilhan sa pamilihan)*  8=Stopped buying because no money available to buy *(Hindi na bumibili ng klase ng pagkain na ito dahil walang pambili)*  9= Stopped buying because receive aids from government or other sources *(Hindi na bumibili ng pagkain na ito dahil nakatanggap mula sa gobyerno/iba pa)*  10=Stopped buying for other reasons *(Hindi na bumibili ng klase ng pagkain na ito dahil sa iba pang dahilan)* | | | | |  |
| **5.1** | | **5.1.a** | Rice (bigas) | \|__\| | | | | | \|__\| | | | | | |
|  |  | **5.1.b** | Bread (Tinapay) | \|__\| | | | | | \|__\| | | | | | |
|  |  | **5.1.c** | Tubers (Lamang lupa katulad ng patatas, kamote) | \|__\| | | | | | \|__\| | | | | | |
|  |  | **5.1.d** | Corn (mais) | \|__\| | | | | | \|__\| | | | | | |
|  |  | **5.1.e** | Noodle/Pasta *(pansit)* |  | | | | |  | | | | | |
| **5.2** | | Beans, pulse, lentils, chickpeas, tofu *(Beans, munggo, kidney beans, garbanzos, tokwa*) | | \|__\| | | | | | \|__\| | | | | | |
| **5.3** | | Nuts and seeds *(mani, kasuy, pistachio, butong pakwan)* | | \|__\| | | | | | \|__\| | | | | | |
| **5.4** | | Dairy, milk, yogurt *(produktong gawa sa gatas tulad ng, keso, gatas, ice cream, yogurt)* | | \|__\| | | | | | \|__\| | | | | | |
| **5.5** | | Breastmilk substitutes / infant formula *(mga pamalit sa gatas ng ina/ mga komersyal na gatas para sa sanggol)* | | \|__\| | | | | | \|__\| | | | | | |
| **5.6** | | **5.6.a** | Meat (karne ng baboy at baka) | \|__\| | | | | | \|__\| | | | | | |
|  |  | **5.6.b** | Poultry (karne ng manok) | \|__\| | | | | | \|__\| | | | | | |
|  |  | **5.6.c** | Fish (Isda) | \|__\| | | | | | \|__\| | | | | | |
|  |  | **5.6.d** | Seafood (hipon, alimasag, tahong, atbp) | \|__\| | | | | | \|__\| | | | | | |
|  |  | **5.6.e** | Organ meats (lamang loob) | \|__\| | | | | | \|__\| | | | | | |
| **5.7** | | Eggs *(itlog ng manok, itik, pugo*) | | \|__\| | | | | | \|__\| | | | | | |
| **5.8** | | Vegetables *(Gulay)* | | \|__\| | | | | | \|__\| | | | | | |
| **5.9** | | Fruits *(Prutas)* | | \|__\| | | | | | \|__\| | | | | | |
| **5.10** | | Ready to eat meals *(Lutong pagkain)* | | \|__\| | | | | | \|__\| | | | | | |

| **SECTION 6. Food Insecurity Experience Scale (FIES) (8 QUESTIONS)** | | | | | | | | |
| --- | --- | --- | --- | --- | --- | --- | --- | --- |
| **6.0** –  **Food Insecurity Experience Scale (FIES) (30 days’ recall)**  Now I would like to ask questions about your experience on food. During the last 30 days, was there a time when:  *Ngayon naman po ay tatanungin ko po kayo ng patungkol sa mga karanasan ninyo sa pagkain. Sa nakaraang 30 araw, may pagkakataon po bang:*  [READ EACH ITEM AND LET THE RESPONDENT ANSWER] | | | | | | 1=Yes *(Oo)*  2=No *(Hindi)*  98=Do not know *(Hindi alam)*  97=Do not want to answer *(Ayaw sumagot)* | | |
| **6.1** | Was there a time when you or other members in your household were worried about not having enough food to eat because of lack of money or other resources?  *May pagkakataon po ba na kayo o miyembro ng inyong sambahayan ay nag-alala dahil sa kakulangan ng pagkain dulot ng kakulangan ng pera o mapagkukunan?* | | | | | \|___\| | | |
| **6.2** | Was there a time when you or other members in your household were unable to eat healthy and nutritious foods because of lack of money or other resources?  *May pagkakataon po ba na kayo o miyembro ng inyong sambahayan ay hindi nakakain ng pagkaing masustansya (katulad ng prutas, gulay, sariwang karne ng baboy/manok/baka, atbp.) dahil sa kakulangan ng pera at iba pang mapagkukunan?* | | | | | \|___\| | | |
| **6.3** | Was there a time when you or other members in your household ate only few kinds of food because of lack of money or other resources?  *May pagkakataon po ba na kayo o ibang miyembro ng inyong sambahayan ay kumain ng iilang uri ng pagkain lamang dahil sa kakulangan sa pera o iba pang mapagkukunan?* | | | | | \|___\| | | |
| **6.4** | Was there a time when you or other members in your household skip a meal because of lack of money or other resources?  *May pagkakataon po ba na kayo o ibang miyembro ng inyong sambahayan ay lumaktaw ng pagkain dahil walang sapat na pera o ibang mapagkukunan ng pagkain?* | | | | | \|___\| | | |
| **6.5** | Was there a time when you or other members in your household ate less than you thought you should because of lack of money or other resources?  *May pagkakataon po ba na kayo o ibang miyembro ng inyong sambahayan ay kumain ng kaunti kaysa sa naiisip niyong tamang dami dahil sa kakulangan sa pera o ibang mapagkukunan?* | | | | | \|___\| | | |
| **6.6** | Was there a time when you or other members in your household ran out of food because of lack of money or other resources?  *May pagkakataon po ba na kayo o ibang miyembro ng inyong sambahayan ay naubusan ng pagkain dahil sa kakulangan sa pera o iba pang mapagkukunan?* | | | | | \|___\| | | |
| **6.7** | Was there a time when you or other members in your household were hungry but did not eat food because of lack of money or other resources?  *May pagkakataon po ba na kayo o ibang miyembro ng inyong sambahayan ay nagutom ngunit hindi kumain dahil walang sapat na pera o mapagkukunan ng pagkain?* | | | | | \|___\| | | |
| **6.8** | Was there a time when you or other members in your household went without eating for a whole day because of lack of money or other resources?  *May pagkakataon po ba na kayo o ibang miyembro ng inyong sambahayan ay hindi kumain ng isang buong araw dahil sa kakulangan sa pera o ibang mapagkukunan?* | | | | | \|___\| | | |
| **SECTION 7. Water, Sanitation, And Hygiene [WASH] (4 questions)** | | | | | | | |  |
| **7.1** | | In the past week, do you have enough soap and water to wash your hands as needed?  *Noong nakaraang linggo, kayo po ba ay may sapat na sabon at tubig para mag hugas ng kamay kung kinakailangan?* | | 1=Yes, sufficient *(Oo, sapat naman)*  => **Proceed to** **7.3**  0=No, insufficient *(Wala, hindi sapat)* | | | \|___\| |  |
| **7.2** | | What is the main reason you or members of your household do not have enough soap and water for your needs?  *Bakit po wala kayong sapat na sabon at tubig?*  [DO NOT READ THE CHOICES. LET THE RESPONDENT ANSWER] | | 1= Unable to access water *(walang mapag kukunan ng tubig)*  2=Water supply reduced *(nabawasan ang supply ng tubig)*  3=Unable to access soap *(walang mapag kukunan ng sabon)*  4=Afraid of going out and getting the virus *(natatakot lumabas at mahawahan ng virus)*  5=Others, specify ____________________ | | | \|___\| |  |
| **7.3** | | Has the consumption of changed during COVID-19 pandemic? *(May pagbabago ba sa konsumo ng mga sumusunod dahil sa COVID-19 pandemic?)*  1 = yes, more *(oo, tumaas)*  2 = yes, less *(oo, bumaba)*  3 = no, the same *(wala)*  4 = doesn’t apply  5 = doesn’t know *(hinid alam)* | | 1. water (domestic use) 2. drinking water 3. soap 4. alcohol 5. other disinfectants (bleach or chlorine solutions) | | | 1. \|___\| 2. \|___\| 3. \|___\| 4. \|___\| 5. \|___\| |  |
| **7.4** | | Have you received reminders and messages related to hand washing and hygiene specifically to avoid the threat of Covid19? For example, SMS, social media platforms, radio, TV, press  *Kayo po ba ay nakatanggap ng mga mensahe tungkol sa paghuhugas ng kamay at kalinisan partikular sa pag-iwas sa banta ng Covid19? halimbawa: text messages, social media, radyo, at telebisyon.* | | 1 = Yes *(Oo)*  0 = No *(Hindi)* => **Proceed to 8.1** | | | \|___\| |  |
| **7.5** | | What are your trusted sources of news and/or reminders (about WASH)?  *Ano po ang mga pinagkakatiwalaan nyong mapagkukunan ng mga balita at paalala (patungkol sa WASH)?*  [DO NOT READ THE CHOICES. LET THE RESPONDENT ANSWER]  0 = No *(Hindi)*  1 = Yes*(Oo)* | | 1. TV, radio  2. newspaper, flyer, brochure  3. banner, billboard  4. Social media (FB, IG, Twitter)  5. Whatsapp  6. SMS (text)  7. Informal network (person to person)  8. Others, specify _____________________ | | | \|___\|  \|___\|  \|___\|  \|___\|  \|___\|  \|___\|  \|___\|  \|___\| |  |
| **7.6** | | What does this household use for disposal of human urine and feces?  *“Ano ang kadalasan ninyong ginagamit na palikuran?”* | | 1=Flush toilet (to piped sewer, septic tank, or pit latrine)  2=pit latrine with slab  3=Pit latrine without floor/slab  4=No facility, field, bush, plastic bag  98=Don’t know  Other, specify _________________ | | |  |  |
| **7.7** | | Do you share your sanitation facility with other households?  “*May iba pa po bang nakikigamit ng inyong palikuran bukod sa inyong pamilya?”* | | 1=Yes, shared with neighbors (small number of households who know each other  2= Yes, shared with neighbors (small number of households who know each other  3=No, private toilet  4=No toilet  98=Don’t know  Other, specify _________________ | | |  |  |
| **7.8** | | What is the ***main*** source of drinking water for your household?  *“Ano ang pangunahing pinagkukuhanan ninyo ng tubig para sa inumin?”*  DO NOT READ THE ANSWERS. SELECT ONE PRIMARY SOURCE ONLY. | | 01=Piped water into dwelling  02=Public tap/standpipe  03=Tubewell/borehole (& pump)  04=Dug well/spring  05=Rain water collection  06=Packaged Water (bottle or sachet)  07-Delivered water (Tanker truck)  08=Water kiosk  09=Surface water (river, stream, dam, lack, pond, canal,  10=Don’t know  Other, specify ____________ | | |  |  |
| **7.9** | | In the last week, where did your household collect water from? *“Nitong nakaraang linggo, saan kayo kumuha ng tubig?* | | 01=In own dwelling  02= In own yard or plot  03=Elsewhere  04=Don’t know  Other, specify ____________ | | |  |  |
| **Section 8 – COVID-19 Shock (1 QUESTION – 14 ITEMS)** | | | | | | | |  |
| What are your concerns in the current situation?  *Ano po ang mga pinag aalala ninyo sa kasalukuyang mga pangyayari?*  [DO NOT READ OUT THE RESPONSE OPTION. LET THE RESPONDENT ANSWER.] | | | | | Mentioned by household member  0 = No *(Hindi)*  1 = Yes *(Oo)* | | |  |
| **8.1** | | | No concerns *(Walang alalahanin)* | | \|__\| | | |  |
| **8.2** | | | Shortage of food *(Kakulangan sa pagkain)* | | \|__\| | | |  |
| **8.3** | | | Increase in food prices *(Pagtaas ng presyo ng pagkain)* | | \|__\| | | |  |
| **8.4** | | | Shortage of medicine *(Kakulangan ng gamot)* | | \|__\| | | |  |
| **8.5** | | | Disruption of medical service *(Pagkagambala ng serbisyong medical)* | | \|__\| | | |  |
| **8.6** | | | Disruption of educational institutes *(Pagkagambala ng mga institusyong pang edukasyon)* | | \|__\| | | |  |
| **8.7** | | | Getting sick *(Pag kakasakit)* | | \|__\| | | |  |
| **8.8** | | | Lack of work *(kakulangan ng trabaho)* | | \|__\| | | |  |
| **8.9** | | | Disruption of livelihood source *(Pagka antala ng mapagkukunan ng kabuhayan)* | | \|__\| | | |  |
| **8.10** | | | Travel restrictions *(Pag hihigpit sa pag lalakbay)* | | \|__\| | | |  |
| **8.11** | | | Paying the rent *(Pag babayad ng upa)* | | \|__\| | | |  |
| **8.12** | | | Paying electricty bills *(Pag babayad ng kuryente)* | | \|__\| | | |  |
| **8.13** | | | Paying water bill *(Pag babayad ng tubig)* | | \|__\| | | |  |
| **8.14** | | | Others, specify____________________ | | \|__\| | | |  |

| **SECTION 9 – FOLLOW UP (3 QUESTIONS)** | | |
| --- | --- | --- |
| Do you belong to the group of informal settlers?  *Kayo po ba ay kabilang sa informal settlers?* |  |  |
| We will conduct another short interview next month, would you be willing to provide us additional information in the follow-up survey?  *Muli kaming magsasagawa ng maikling panayam sa susunod na buwan. Kayo po ba ay sumasangayon na magbigay ng mga karagdagang impormasyon sa aming follow-up survey?* | 0 = No *(Hindi)*  1 = Yes *(Oo)* | \|____\| |
| In case we cannot reach you on this phone number in a month’s time, are you able to provide us a back-up number from a family member or friends?  *Sa pagkakataong hindi namin kayo matawagan sa iyong numero sa susunod na buwan ay maaari po ba kayong magbigay ng isa pang numero ng miyembro ng inyong tahanan?* |  | |
| If yes, what is the preferred time for receiving a call from us?  *Ano po ang pinakamagandang oras na pwede po namin kayo matawagan ulit?* | 1= Anytime (9:00-20:00)  2=Morning (9:00-12:00)  3=Afternoon (12:00-16:00)  4=Evening (16:00-20:00) | \|____\| |

READ TO THE RESPONDENT:

Thank you very much for the interview. As a token of appreciation, we will now transfer [amount] to this phone number. Have a great day.

*Maraming salamat po sa inyong partisipasyon sa aming panayam. Bilang aming pasasalamat, aming bibigyan ng [amount] load ang numerong ito. Magandang Araw po!*

**Urban study research REMOTE “Status and determinants of food insecurity and undernutrition in poor urban areas” (SDFU-2020)**

**MOTHER AND U5 CHILDREN SURVEY QUESTIONNAIRE (MCQ)**

**(THE QUESTIONNAIRE WILL BE ADMINISTRATED BY PHONE)**

**INFORMED CONSENT PAGE 1**

**SECTION 1 – MOTHER AND YOUNGEST CHILD PROFILE (9Q) PAGE 1**

**SECTION 1A –WOMEN DIET DIVERSITY (1Q-13 ITEMS) PAGE 2**

**SECTION 1B – FOOD PURCHASED FOR WOMEN (2Q) PAGE 3**

**PREGNANT -> SECTION 2A – PNC AND BIRTH INFORMATION (3 Q) PAGE 3**

**GAVE BIRTH SINCE JAN 2020 -> SECTION 2B – PNC AND BIRTH INFORMATION (4 Q) PAGE 4**

**0-23 MONTHS -> SECTION 3A - INFANT AND YOUNG CHILD FEEDING PRACTICES (17Q) PAGE 5**

**24-59 MONTHS -> SECTION 3B – CHILD’S DIET (3Q) PAGE 9**

**SECTION 4 - CHILD ILLNESS AND CARE (3Q) PAGE 10**

**SECTION 5 –FOOD PURCHASED FOR CHILDREN (2Q) PAGE 11**

**Date**|__||__|/|__||__|/|_2_||_0_|**(DD/MM/YY) TIME STARTED: |__||__|:|__||__| TIME ENDED: |__||__|:|__||__|**

| **INFORMED CONSENT (2 QUESTIONS)** | | | | | | | | | | | | | | | | | | | | | | | | | |
| --- | --- | --- | --- | --- | --- | --- | --- | --- | --- | --- | --- | --- | --- | --- | --- | --- | --- | --- | --- | --- | --- | --- | --- | --- | --- |
| **A** | | Just to confirm, are you a mother of a U5 child?  *Linawin ko lang po, kayo po ba ay ina ng batang may edad na limang taon o mababa sa limang taon?* | | | | | | | | | | 0=No *(Hindi)* => **End the survey**  1=Yes *(Oo)* | | | | | | | | | \|___\| | | | | |
| **B** | | Do you agree to participate in our study?  *Sumasang-ayon po ba kayong makilahok sa aming pag-aaral?* | | | | | | | | | | 0=No *(Hindi)* => **End the survey**  1=Yes *(Oo)* | | | | | | | | | \|___\| | | | | |
| **Section 1 – MOTHER AND YOUNGEST CHILD PROFILE (9 QUESTIONS)** | | | | | | | | | | | | | | | | | | | | | | | | | |
| **1.1** | | How old are you?  *Ilang taon na po kayo?* | | | | | \|___\|\|___\| taong gulang | | | | | | | | | | | | | | | | | | |
| **1.2** | | What is your highest educational attainment?  *Ano po ang inyong natapos?* | | | | | 0=None  1=Elementary undergraduate (Grade 1-6)  2=Elementary graduate  3=Highschool undergraduate (Grade 7-10)  4=Highschool graduate  5=Senior Highschool undergraduate (Grade 11-12)  6=Senior Highschool graduate  7=College undergraduate  8=College graduate  9=Post-graduate  10=Technical/Vocational | | | | | | | | | | | | | | | | | \|___\| | |
| **1.3** | | Are you currently pregnant?  *Kayo po ba ay kasalukuyang buntis?* | | | | | 0=No => **Do not answer 2A.1-3**  1=Yes => **Answer 2A.1-3**  98=Do not know | | | | | | | | | | | | | | | | | \|___\| | |
| **1.4** | | Have you given birth since January 2020?  *Kayo po ba ay nanganak pamula pa noong Enero 2020?* | | | | | 0=No => **Do not answer 2B.1-4**  1=Yes => **Answer 2B.1-4** | | | | | | | | | | | | | | | | | \|___\| | |
| **1.5** | | How many U5 children do you have?  *Ilan po ang inyong anak na may edad na limang taon o mababa sa limang taon?* | | | | | \|___\|\|___\| | | | | | | | | | | | | | | | | | | |
| **1.6** | | When was your youngest child born?  *Kailan po ipinanganak ang inyong bunsong anak?*  [calculate number of months based on date of interview] | | | | | \|___\|\|___\| Day  \|___\|\|___\| Month  \|___\|\|___\|\|___\|\|___\| Year  98=Do not know | | | | | | | | | | | | | | | | | | |
| **1.7** | | What is the gender of your youngest child?  *Ano po ang kasarian ng inyong bunsong anak?* | | | | | 1=Male  2=Female | | | | | | | | | | | | \|___\| | | | | | | |
| **1.8** | | Does your youngest child have a twin/ triplet/ quadruplet sibling/s?  *Mayroon po bang kakambal/triplet/quadruplet na kapatid ang inyong bunsong anak?* | | | | | 0 = None *(Wala)* => **Proceed to 1A**  1 = Yes *(Oo)* | | | | | | | | | | | | \|___\| | | | | | | |
| **1.9** | | What is the gender of the twin/triplet/quadruplet sibling/s of your youngest child?  *Ano ang kasarian ng kakambal/triplet/quadruplet na kapatid ng iyong bunsong anak?* | | | | | 1=Male  2=Female | | | | | | | Twin \|___\|  Triplet \|___\|,\|___\|  Quadruplet\|___\|,\|___\|,\|___\| | | | | | | | | | | | |
| **SECTION 1A – Women diet diversity (check that the respondent is aged 15 to 49 years old) (1 QUESTION – 13 ITEMS)** | | | | | | | | | | | | | | | | | | | | | | | | | |
| Now I’d like to ask you about foods and drinks that you ate or drank yesterday during the day or night, whether you ate it at home or anywhere else. Think about all the food you ate yesterday after you woke up in the morning, in the afternoon and at night. Please include all foods and drinks, any snacks or small meals, as well as any main meals.  *Ngayon tatanungin ko naman po kayo ng mga kinain at ininom ninyo pamula kahapon ng umaga hanggang gabi, kasama ang mga kinain mo sa loob at labas ng bahay. Isipin nyo po lahat ng kinain nyo simula nang magising kayo kahapon ng umaga hanggang bago matulog.*  What did you eat or drink in the morning after you woke up?  *Ano po ang inyong kinain at ininom pagkagising kahapon ng umaga? sa tanghali?*  What did you eat or drink during the afternoon?  *Ano po ang inyong kinain at ininom kahapon ng hapon?*  What did you eat or drink during the evening/night before going to bed?  *Ano po ang inyong kinain at ininom kagabi hanggang bago kayo matulog?*  [IF THE RESPONDENT MENTIONS A MIXED DISH, ASK FOR ALL THE INGREDIENTS IN THE MIXED DISH. CONTINUE TO PROBE ABOUT INGREDIENTS UNTIL SHE SAYS “NOTHING ELSE”. AS THE RESPONDENT RECALLS FOODS AND DRINKS, CORRECTLY CLASSIFY THE REPORTED FOODS INTO THE FOOD GROUPS; SELECT ALL THE RELEVANT FOOD GROUPS.] | | | | | | | | | **[DO NOT READ THE FOOD GROUPS]**  **A**  What food groups have you eaten for past 24 hours (day and night)?  *Ano-anong grupo ng pagkain po ang inyong nakain sa nakalipas na 24h (umaga at gabi)?*  0=No *(Hindi)*  1=Yes *(Oo)*  98= Do not know *(Hindi alam)* | | | | | | | | | **[READ THE FOOD GROUPS]**  **B**  Has your consumption change because of Covid 19 pandemic?  *Nag bago po ba ang inyong pag konsumo ng mga pagkaing ito dahil sa Covid19?*  1=Yes, increase consumption  2=Yes, decrease consumption  3=No change | | | | | | | |
| **1A.1** | **1A.1a** | | | | Rice *(bigas)* | | | | \|___\| | | | | | | | | | \|___\| | | | | | | | |
|  | **1A.1b** | | | | Bread *(tinapay)* | | | | \|___\| | | | | | | | | | \|___\| | | | | | | | |
|  | **1A.1b** | | | | Tubers *(lamang lupa katulad ng patatas, kamote)* | | | | \|___\| | | | | | | | | | \|___\| | | | | | | | |
|  | **1A.1d** | | | | Corn *(mais)* | | | | \|___\| | | | | | | | | | \|___\| | | | | | | | |
|  | **1A.1e** | | | | Noodles/Pasta *(pansit)* | | | |  | | | | | | | | |  | | | | | | | |
| **1A.2** | Beans, pulse, lentils, chickpeas, tofu *(Beans, munggo, kidney beans, garbanzos, tokwa*) | | | | | | | | \|___\| | | | | | | | | | \|___\| | | | | | | | |
| **1A.3** | Nuts and seeds *(mani, kasuy, pistachio, butong pakwan)* | | | | | | | | \|___\| | | | | | | | | | \|___\| | | | | | | | |
| **1A.4** | Dairy, milk, yogurt *(produktong gawa sa gatas tulad ng, keso, gatas, ice cream, yogurt)* | | | | | | | | \|___\| | | | | | | | | | \|___\| | | | | | | | |
| **1A.5** | **1A.5a** | | | | Meat (karne ng baboy at baka) | | | | \|___\| | | | | | | | | | \|___\| | | | | | | | |
|  | **1A.5a** | | | | Poultry (karne ng manok) | | | | \|___\| | | | | | | | | | \|___\| | | | | | | | |
|  | **1A.5a** | | | | Fish (Isda) | | | | \|___\| | | | | | | | | | \|___\| | | | | | | | |
|  | **1A.5a** | | | | Seafood (hipon, alimasag, tahong, atbp) | | | | \|___\| | | | | | | | | | \|___\| | | | | | | | |
|  | **1A.5a** | | | | Organ meats (lamang loob) | | | | \|___\| | | | | | | | | | \|___\| | | | | | | | |
| **1A.6** | Eggs *(itlog ng manok, itik, pugo*) | | | | | | | | \|___\| | | | | | | | | | \|___\| | | | | | | | |
| **1A.7** | Dark green leafy vegetables *(berde at madahon na gulay)* | | | | | | | | \|___\| | | | | | | | | | \|___\| | | | | | | | |
| **1A.8** | Other vitamin A rich fruits and vegetables (carrot, pumpkin, orange sweet potato, mango, papaya, dark green leafy vegetables, long beans *(Iba pang prutas at gulay na mayaman sa bitamina A)* | | | | | | | | \|___\| | | | | | | | | | \|___\| | | | | | | | |
| **1A.9** | Other vegetables (eggplant, onion, cucumbers, tomatoes) *(Iba pang gulay katulad ng talong, sibuyas, pipino, at kamatis)* | | | | | | | | \|___\| | | | | | | | | | \|___\| | | | | | | | |
| **1A.10** | Other fruits (banana, apples, pineapple, watermelon) | | | | | | | | \|___\| | | | | | | | | | \|___\| | | | | | | | |
| **1A.11** | Sugar food consumption (chocolate bars, candy bars, candies, sugar-coated food, ice cream, honey, jam, candy, biscuits, pastries, cakes and other sweet products) *(Matatamis na pagkain)* | | | | | | | | \|___\| | | | | | | | | | \|___\| | | | | | | | |
| **1A.12** | Sugary drinks (carbonated soft drink, sweetened tea, sweetened coffee *(Matatamis na inumin)* | | | | | | | | \|___\| | | | | | | | | | \|___\| | | | | | | | |
| **1A.13** | Savory and fried snacks (street foods) *(malinamnam at pritong pagkain katulad ng kwek-kwek, fishball, turon, mga inihaw, atbp)* | | | | | | | | \|___\| | | | | | | | | | \|___\| | | | | | | | |
| **1A.14** | Others (i.e., chichirya/mantika/asin bilang ulam), specify: _____ | | | | | | | | \|___\| | | | | | | | | | \|___\| | | | | | | | |
| **SECTION 1B – FOOD PURCHASED BY WOMEN (2 QUESTIONS)** | | | | | | | | | | | | | | | | | | | | | | | | | |
| **1B.1** | | Yesterday, did you purchase prepared or commercially produced meal for your own consumption?  *Bumili po ba kayo kahapon ng lutong pagkain para sa inyong sarili?* | | | | | | | 0=No *(Hindi)*  1=Yes *(Oo)*  98=Do not know *(Hindi alam)* | | | | | | | | | | | | | \|___\|\|___\| | | | |
| **1B.2** | | Has the expenditure on commercially produced meal changed because of Covid 19 pandemic?  *Nagkaroon po ba ng pagbabago sainyong pagbili ng mga lutong pakain dahil sa Covid19 pandemic? (This also refers to the quarantine)* | | | | | | | 1=Yes, I spend more money  *(Oo, gumastos ng mas maraming pera)*  2=Yes, I spend less money  *(Oo, gumastos ng mas konting pera)*  0=No *(Hindi)*  98=Do not know *(Hindi alam)* | | | | | | | | | | | | | \|___\|\|___\| | | | |
| **SECTION 2 – PNC AND BIRTH INFORMATION (7 QUESTIONS) – only if birth since January 2020 or pregnant** | | | | | | | | | | | | | | | | | | | | | | | | | |
| **[SECTION 2A - IF REPORTED PREGNANT Q1.3=1, FILL IN THIS SECTION]** | | | | | | | | | | | | | | | | | | | | | | | | | |
| **2A.1** | | How many weeks pregnant are you?  *Ilang linggo na po kayong buntis?* | | | | | | \|___\|\|___\| weeks | | | | | | | | | | | | | | | | | |
| **2A.2** | | How many times have you had pre-natal check-ups for this pregnancy?  *Ilang beses na po kayong nagpa pre-natal checkup (PNC) para sa pagbubuntis na ito?*  [FOR THOSE WITH ABOVE AVERAGE NUMBER OF TIMES OF PNC (>8 times), ASK WHAT COULD BE THE REASON FOR SUCH] | | | | | | \|___\|\|___\| times  Reason for above average number of times of PNC: _______________________________________________ | | | | | | | | | | | | | | | | | |
| **2A.3** | | Were there any occasions in which you did not attend a scheduled PNC visit due to COVID19?  *May pagkakataon po bang hindi kayo nakapunta sa nakaschedule niyong PNC dahil sa COVID19?*  [READ THE OPTIONS] | | | | | | 1=No *(Hindi)*  2=Yes, appointment cancelled  *(Oo, nakansela ang appointment)*  3=Yes, appointment postponed  *(Oo, pinagpaliban ang appointment)*  4=Yes, refused to attend due to C19 risk  *(Oo, hindi pumunta dahil sa banta ng Covid19)*  5=Yes, impossible to move and attend PNC *(Oo, imposibleng makapunta para sa PNC)*  96=Not applicable | | | | | | | | | | | | | | | | \|___\| | |
| **[SECTION 2B - IF REPORTED BIRTH SINCE JANUARY 2020 Q1.4=1, FILL IN THIS SECTION]** | | | | | | | | | | | | | | | | | | | | | | | | | |
| **2B.1** | | Who assisted the delivery of (name)? Probe: anyone else?  *Sino po ang nagpaanak sa inyo kay (pangalan ng bata)?*  [PROBE FOR THE TYPE OF PERSON ASSISTING AND CHOOSE ALL ANSWERS GIVEN.]  [IF RESPONDENT SAYS NO ONE ASSISTED, PROBE TO DETERMINE WHETHER ANY ADULTS WERE PRESENT AT THE DELIVERY.]  0 = No  1 = Yes | | | | | | | 1=Health professional *(Mga propesyonal sa kalusugan)*  2=Traditional birth attendant *(mga tradisyonal na nagpapaanak)*  3=Community health worker  4=Relative / Friend *(kamaganak/kaibigan)*  5=Others, please specify _______________  6=No one, any adults present, specify? ________________________ | | | | | | | | | | | | | | | \|___\|  \|___\|  \|___\|  \|___\|  \|___\|  \|___\| | |
| **2B.2** | | How many times did you receive pre-natal care during this pregnancy?  *Ilang beses po kayo nakatanggap ng PNC sa pagbubuntis na ito?*  [PROBE TO IDENTIFY THE NUMBER OF TIMES ANTENATAL CARE WAS RECEIVED. IF A RANGE IS GIVEN, RECORD THE MINIMUM NUMBER OF TIMES ANTENATAL CARE RECEIVED.]  [FOR THOSE WITH ABOVE AVERAGE NUMBER OF TIMES OF PNC (>8 times), ASK WHAT COULD BE THE REASON FOR SUCH] | | | | | | | Actual number of PNC times ____  0=No PNC  98=Do not know | | | | No of times \|__\|\|__\|  DK\|__\|\|__\|  Reason for above average number of times of PNC:  __________________________ | | | | | | | | | | | | |
| **2B.3** | | How often did you take IFA supplements in your last pregnancy?  *Gaano po kayo kadalas uminom ng iron-folic acid (IFA) supplements sa iyong nakaraang pagbubuntis?* | | | | | | | 1=Daily *(araw-araw)*  2=From time to time weekly  *(paminsan-minsan sa isang linggo)*  3=From time to time monthly *(paminsan-minsan sa isang buwan)*  4=Rarely *(Bihira)*  5=Never *(Hindi uminom)* | | | | | | | | | | | | | | \|___\| | | |
| **2B.4** | | How much did (name) weigh in kg at birth?  *Gaano po kabigat si (pangalan ng bata) noong siya ay ipinanganak?* | | | | | | | 1= <2.5 kg  2= >= 2.5 kg  98=Do not know | | | | | | \|___\|\|___\|  If known, specify actual weight  \|__\|.\|__\| kg | | | | | | | | | | |
| **SECTION 3A – INFANT AND YOUNG CHILDREN FEEDING PRACTICES + IMMUNIZATION (16 QUESTIONS)** | | | | | | | | | | | | | | | | | | | | | | | | | |
| **ONLY FOR CHILDREN AGED 0 – 23 MONTHS, IF THE CHILD IS MORE THAN 23 MONTHS PROCEED TO SECTION 3B** | | | | | | | | | | | | | | | | | | | | | | | | | |
| **3A.1** | | Has (NAME) ever been breastfed?  *Si (pangalan ng bata) po ba ay inyong napasuso?* | | | | 0 = No => **Proceed to 3A.7**  1 = Yes  98=Do not know => **Proceed go to 3A.4** | | | | | | | | | | | | | | | | | | \|___\|\|___\| | |
| **3A.2** | | How long after birth was (NAME) first put to the breast?  *Pagka-panganak ninyo po kay (pangalan ng bata), gaano po katagal siya bago nailagay sa inyong dibdib?* | | | | 0 = Immediately or <60 minutes  1=no of hours= >1 hour but <24 hours  2=no of days >24 hours  98 = Do not know | | | | | | | | | | | | | | | | | | \|___\|\|___\| | |
| **3A.3** | | Is (name) still being breastfed?  *Si (pangalan ng bata) po ba ay kasalukuyan ninyo* *pa ring pinapasuso?* | | | | 0=No => **Proceed to 3A.7**  1=Yes  98=Do not know | | | | | | | | | | | | | | | | | | \|___\|\|___\| | |
| **3A.4** | | Was (NAME) breastfed yesterday (day or night)?  *Napasuso nyo po ba si (pangalan ng bata) ano mang oras kahapon?* | | | | **0 =** No => **Proceed to 3A.9**  **1 =** Yes  **98 =** Don’t know => **Proceed to 3A.9** | | | | | | | | | | | | | | | | | | \|___\|\|___\| | |
| **3A.5** | | How many times was (name) breastfed yesterday in the day or in the night?  *Ilang beses nyo pong pinasuso si (pangalan) kahapon?* | | | | \|___\|\|___\| times | | | | | | | | | | | | | | | | | | | |
| **3A.6** | | Has this frequency change because of Covid-19 pandemic?  *Nabago po ba ang dalas ng pagpasuso ninyo kay ______ dahil sa covid19 pandemic?* | | | | 1=Yes, increase *(Oo, mas dumalas)*  2=Yes, decrease *(Oo, mas dumalang)*  0=No *(Hindi)*  98=Do not know *(Hindi alam)*  ***JUMP TO 3A.9.*** | | | | | | | | | | | | | | | | | | \|___\|\|__\| | |
| **3A.7** | | What was the reason for stopping to breastfeed?  *Bakit po kayo tumigil sa pagpapasuso?*  [DO NOT READ THE CHOICES. LET THE MOTHER ANSWER] | | | | 0=Mother mentions Covid19 reasons *(ang dahilan ay may kinalaman sa Covid19)* => Proceed to 3A.5  1=Mother sick/weak *(Mahina or may sakit ang ina)*  2=Child sick/weak *(Mahina or may sakit ang bata)*  3=Breast problems *(May problema sa suso)*  4=Not enough milk *(Walang sapat na gatas)*  5=Mother works *(Nagtatrabaho ang ina)*  6=Child refused breast *(Ayaw sumuso ng bata)*  7=Weaning age *(Nag simula na kumain ng solid foods ang bata)*  8=New pregnancy *(Kasalukuyang buntis)*  9=Started using contraceptives *(Nagsimula gumamit ng contraceptives)*  10=Other, specify __________________________ | | | | | | | | | | | | | | | | | | \|___\|\|___\| | |
| **3A.8** | | If you stopped breastfeeding because of COVID related reasons, why was this?  *Bakit po ninyo itinigil ang pag papasuso kay ___ dahil sa Covid 19?*  [MULTIPLE ANSWERS POSSIBLE. DO NOT READ THE CHOICES. LISTEN AND TICK THE ONES THAT ARE REPORTED]  Then **Proceed to 3A.9** | | | | 1=Doctor/nurse told me to stop breastfeeding or not to start breastfeeding *(Sinabihan ako ng Doktor/Nars na itigil ko ang aking pagpapasuso or wag mag pasuso)*  2=A family member/friend told me to stop/not to breastfeed  *(Sinabihan ako ng aking pamilya or kaibigan na itigil ko ang aking pagpapasuso or wag mag pasuso)*  3=I heard on the radio/TV/read online that Covid is in breastmilk and not to breastfeed *(Nabalitaan ko sa TV/radio/nabasa online na ang COVID ay nasa gatas ng ina at wag magpasuso)*  4=I heard on social media that covid is in Breastmilk and not to breastfeed  *(Nabalitaan ko sa socal media na ang COVID ay nasa gatas ng ina at wag mag pasuso)*  5=I heard marketing messages from formula companies that it is better to formula feed in the context of Covid  *(Nabalitaan ko sa patalastas ng mga kumpanya ng gatas na mas maganda ipainom ng formula milk ngayong may Covid)*  6=I am now separated with my child  *(Hiwalay na ako sa aking anak)*  7=I was given free formula milk  *(Nabigyan ako ng libreang formula milk)*  8=I just decided i did not want to breastfeed  *(Napagdesisyunan ko na ayoko na mag pasuso)*  9=Other reasons, specify _____________________  98=Do not know *(Hindi alam)* | | | | | | | | | | | | | | | | | | \|___\|  \|___\|  \|___\|  \|___\|  \|___\|  \|___\|  \|___\|  \|___\|  \|___\|  \|___\| | |
| **3A.9** | | What are the ways you know that can increase milk production?  *Ano po ang mga paraan na alam ninyo na makakapag padami ng gatas ng ina?* | | | | 0=None *(wala)*  1=Eat more green leafy vegetables *(pag kain ng mas maraming madadahong gulay i.e. malunggay)*  2=Eating viands with soup *(pag kain ng ulam na may sabaw)*  3=Drink more water *(pag inom ng mas maraming tubig)*  4=Breastfeeding the child more frequently *(pag papasuso sa anak ng mas madalas)*  5=Pumping milk regularly *(regular na pag-pump ng gatas)*  6=Taking lactation supplements *(pag inom ng mga supplements na nakakapag-padami ng gatas*  7=Others, specify ________________________________ | | | | | | | | | | | | | | | | | | \|___\| | |
| **3A.10** | | Yesterday, during the day or night did (name) _______, drink or eat vitamins or mineral supplements, drops or any medicines?  *Uminom po ba ng kahit anong bitamina o gamot si (pangalan) kahapon?* | | | | 1=Yes *(Oo)*  0=No *(Hindi)*  98=Do not know *(Hindi alam)* | | | | | | | | | | | | | | | | | | \|___\|\|___\| | |
| **3A.11** | | Now I want to ask you what **liquids** (NAME) received yesterday during the day and night – even if combined with other foods, include liquids consume outside of your home  *Ngayon tatanungin ko naman po kayo sa mga ininom ni (pangalan) kahapon ng umaga at gabi kasama ang tubig o iba pang inumin na idinagdag sa pagkain at mga kinain sa labas ng bahay.* | | | | | | | | | | | | | | | | | | | | | | | |
|  |  | Did (Name) drink: Si (pangalan) ba ay uminom ng: | | | | | | | | | | | | | | | | | | **0**= No  **1**= Yes  **98**= Don’t know | | | | | |
|  |  | **3A.11a.** Plain water? *(inuming tubig)* | | | | | | | | | | | | | | | | | | \|__\|\|__\| | | | | | |
|  |  | **3A.11b.** Juice or juice drinks? | | | | | | | | | | | | | | | | | | \|__\|\|__\| | | | | | |
|  |  | **3A.11c.** Clear soup / broth? *(sabaw)* | | | | | | | | | | | | | | | | | | \|__\|\|__\| | | | | | |
|  |  | **3A.11d.** Artificial milk for babies (infant/baby formula or follow on/growing up formula serving as breast milk substitutes, ex Dumex, Guigoz, Lactogen, etc) *(artipisyal na gatas ng bata)* | | | | | | | | | | | | | | | | | | \|__\|\|__\| | | | | | |
|  |  | **3A.11d.1** number of times drunk artificial milk? (if unknown, record ‘99’) *(dami ng beses na uminom ng artipisyal na gatas ang bata)* | | | | | | | | | | | | | | | | | | \|__\|\|__\| | | | | | |
|  |  | **3A.11e.** Any other type of milk (any type of animal milk powdered, fresh, UHT but not sweetened condensed milk or baby formula; may also ask if child was given reconstituted coffee creamer, evaporated milk, soy milk)? *(iba pang uri ng gatas)* | | | | | | | | | | | | | | | | | | \|__\|\|__\| | | | | | |
|  |  | **3A.11e.1** number of times drunk other type of milk? (if 7 or more times, record ‘7’, if unknown, record ‘8’) *(dami ng beses na uminom ng iba pang uri ng gatas ang bata)* | | | | | | | | | | | | | | | | | | \|__\|\|__\| | | | | | |
|  |  | **3A.11g.** Tea or coffee? *(Tsaa or kape?)* | | | | | | | | | | | | | | | | | | \|__\|\|__\| | | | | | |
|  |  | **3A.11h.** Rice water? *(Am?)* | | | | | | | | | | | | | | | | | | \|__\|\|__\| | | | | | |
|  |  | **3A.11i.** Sugar sweetened beverages (soft drinks, sweetened tea)? *(matatamis na inumin?)* | | | | | | | | | | | | | | | | | | \|__\|\|__\| | | | | | |
| **3A.12** | | Has the type of liquid given changed because of Covid 19 pandemic?  *Nagbago po ba ang uri ng inumin na binibigay ninyo kay ____ dahil sa covid19 pandemic?* | | | | | | | | 0=No *(Hindi)*  1=Yes, more of certain types *(Oo, mas maraming uri)*  2=Yes, less of certain types *(Oo, mas kaunting uri)*  98=Do not know *(Hindi alam)* | | | | | | | | | | \|__\|\|__\| | | | | | |
| **3A.13** | | Now I would like to ask you about everything that (name) ate yesterday during the day or the night. Please include foods consumed outside of your home.  *Ngayon po ay tatanungin ko naman kayo ng patungkol sa lahat ng kinain ni (pangalan) kahapon pamula umaga hanggang gabi. Pakisama din lahat ng pagkain na kinain sa labas ng inyong bahay.*  Think about when (name) woke up yesterday. Did (he/she) eat anything at that time? If ‘Yes’ ask: Please tell me everything (name) ate at that time. Probe: Anything else?  *Isipin po ninyo kung ano ang mga kinain ni (pangalan) noong pagkagising niya kahapon ng umaga. Mayroon pa po bang iba?*  What did (name) do after that? Did (he/she) eat anything at that time?  *Ano po ang mga ginawa ni (pangalan) pagkatapos noon? May kinain pa po ba sya noon?*  [Repeat this string of questions, recording in the food groups, until the respondent tells you that the child went to sleep until the next morning] | | | | | | | | | | | | | | | | | | | | | | | |
|  |  | Did (Name) eat:  *Si (pangalan) ba ay kumain ng:*  [FOR COLUMN A, DO NOT READ THE FOOD GROUPS]  [FOR COLUMB B, READ THE FOOD GROUPS] | | | | | | | **A**  **0**= No *(Hindi)*  **1**= Yes *(Oo)*  **98**= Don’t know *(Hindi alam)* | | | | | **B**  Has (name) consumption change because of Covid19 pandemic?  *Nagbago po ba ang konsumo ni (name) dahil sa Covid19?*  1=Yes, increase consumption *(Oo, tumaas ang konsumo)*  2=Yes, decrease consumption *(Oo, bumaba ang konsuma)*  3=No change | | | | | | | | | | | |
|  |  | **3A.13a.** Any commercially fortified baby food (Cerelac, Gerber, NutriLove, Happy Baby Cereal, Milna, Health Times, Baby Natura)? | | | | | | | \|__\|\|__\| | | | | | \|__\|\|__\| | | | | | | | | | | | |
|  |  | **3A.13b.** | | i. Rice *(bigas)* | | | | | \|__\|\|__\| | | | | | \|__\|\|__\| | | | | | | | | | | | |
|  |  |  |  | ii. Bread *(tinapay)* | | | | | \|__\|\|__\| | | | | | \|__\|\|__\| | | | | | | | | | | | |
|  |  |  |  | iii. Tubers *(lamang lupa katulad ng patatas, kamote)* | | | | | \|__\|\|__\| | | | | | \|__\|\|__\| | | | | | | | | | | | |
|  |  |  |  | iv. Corn *(mais)* | | | | | \|__\|\|__\| | | | | | \|__\|\|__\| | | | | | | | | | | | |
|  |  |  |  | v. Noodle/Pasta *(pansit)* | | | | | \|__\|\|__\| | | | | | \|__\|\|__\| | | | | | | | | | | | |
|  |  | **3A.13c.** Pulses: beans/peas, peanuts, lentils, almond, and/or other pulses | | | | | | | \|__\|\|__\| | | | | | \|__\|\|__\| | | | | | | | | | | | |
|  |  | **3A.13d.** Dairy products: yogurt, cheese, other dairy (exclude margarine/butter or small amounts of milk for tea/coffee) *(produktong gawa sa gatas tulad ng, keso, gatas, ice cream, yogurt)* | | | | | | | \|__\|\|__\| | | | | | \|__\|\|__\| | | | | | | | | | | | |
|  |  | **3A.13e.** | | i. Meat (karne ng baboy at baka) | | | | | \|__\|\|__\| | | | | | \|__\|\|__\| | | | | | | | | | | | |
|  |  |  |  | ii. Poultry (karne ng manok) | | | | | \|__\|\|__\| | | | | | \|__\|\|__\| | | | | | | | | | | | |
|  |  |  |  | iii. Fish (Isda) | | | | | \|__\|\|__\| | | | | | \|__\|\|__\| | | | | | | | | | | | |
|  |  |  |  | iv. Seafood (hipon, alimasag, tahong, atbp) | | | | | \|__\|\|__\| | | | | | \|__\|\|__\| | | | | | | | | | | | |
|  |  |  |  | v. Organ meats (lamang loob) | | | | | \|__\|\|__\| | | | | | \|__\|\|__\| | | | | | | | | | | | |
|  |  | **3A.13f.** Eggs *(itlog)* | | | | | | | \|__\|\|__\| | | | | | \|__\|\|__\| | | | | | | | | | | | |
|  |  | **3A.13g.** Vitamin A rich fruits and vegetables (including dark leafy vegetables) *(prutas at gulay na mayaman sa bitamina A)* | | | | | | | \|__\|\|__\| | | | | | \|__\|\|__\| | | | | | | | | | | | |
|  |  | **3A.13h.** Other vegetables and fruits *(iba pang gulay at prutas)* | | | | | | | \|__\|\|__\| | | | | | \|__\|\|__\| | | | | | | | | | | | |
|  |  | **3A.13i**. Savory and fried snacks (street foods) *(malinamnam at pritong pagkain katulad ng kwek-kwek, fishball, turon, mga inihaw, atbp)* | | | | | | | \|__\|\|__\| | | | | | \|__\|\|__\| | | | | | | | | | | | |
|  |  | **3A.13j**. Sugary foods: chocolate bars, candy bars, candies, sugar-coated food, ice cream, honey, jam, candy, biscuits, pastries, cakes and other sweet products *(matatamis na pagkain)* | | | | | | | \|__\|\|__\| | | | | | \|__\|\|__\| | | | | | | | | | | | |
|  |  | **3A.13k**. Others (i.e. chichirya/mantika/asin bilang ulam), specify: ______________ | | | | | | | \|__\|\|__\| | | | | | \|__\|\|__\| | | | | | | | | | | | |
| **3A.14** | | Yesterday, during the day and night, how many times did (NAME) eat any solid, semisolid or soft foods?  *Ilang beses po kumain ng pagkain si (pangalan) kahapon?* | | | | | | | **0 =** None  **1 =** 1 time  **2 =** 2 times  **3 =** 3 times  **4 =** 4 times  **5 =** 5 times  **6 =** 6 times  **7 =** 7 or more times  **98 =** Do not know  *(Hindi alam)* | | | | | \|__\|\|__\| | | | | | | | | | | | |
| **3A.15** | | Has this frequency of food consumption changed because of Covid 19 pandemic?  *Nagbago ba ang dalas ng pagkain dahil sa covid19?* | | | | | | | 0=No  1=Yes, more frequent  *(Oo, mas madalas)*  2=Yes, less frequent  *(Oo, hindi na madalas)*  98=Do not know  *(Hindi alam)* | | | | | \|__\|\|__\| | | | | | | | | | | | |
| **3A.16** | | Did you hear or receive any message in the last month on breastfeeding of infants in the context of COVID?  *Nakarinig o nakatanggap po ba kayo ng mensahe noong nakaraang buwan patungkol sa pagpapasuso ng mga sanggol ngayong may Covid19 pandemic?*  [Examples of messages might be on breastfeeding, what foods to feed a young child, or how to feed during and after illness. Examples of sources of messages might include: social media (give examples), on a website, radio, television, Facebook, health talk at a health facility or by a community health worker or mother support group, religious institution, poster or leaflet etc.] | | | | | | | 0=No *(Hindi)*  1=Yes *(Oo)*  98=Do not know *(Hindi alam)* | | | | | \|__\|\|__\| | | | | | | | | | | | |
| **3A.17** | | Did you hear or receive any message in the last month on feeding a child aged 6 months to 2 years of age? (complementary feeding)  *Nakarinig o nakatanggap po ba kayo ng mensahe noong nakaraang buwan patungkol sa pagpapakain ng mga sanggol edad 6 na buwan hanggang dalawang taong gulang? (complementary feeding)* | | | | | | | 0=No *(Hindi)*  1=Yes *(Oo)*  98=Do not know *(Hindi alam)* | | | | | \|__\|\|__\| | | | | | | | | | | | |
| **SECTION 3B – CHILDREN DIET 24 MONTHS+ (3 QUESTIONS)** | | | | | | | | | | | | | | | | | | | | | | | | | |
| **ONLY FOR CHILDREN AGED 24 MONTHS AND ABOVE** | | | | | | | | | | | | | | | | | | | | | | | | | |
| **3B.1** | | What are the food groups that your child ate in the past 24h (day and night)?  *Ano-ano pong grupo ng pagkain ang kinain ng inyong anak sa nakaraang 24 oras?*  [DO NOT READ THE FOOD GROUPS. LET THE RESPONDENT ANSWER.] | | | | | | | | | | | | | | 0=No  1=Yes  98=Do not know | | | | | | | | | |
|  |  | **3B.1a** | i. Rice *(bigas)* | | | | | | | | | | | | | \|__\|\|__\| | | | | | | | | | |
|  |  |  | ii. Bread *(tinapay)* | | | | | | | | | | | | | \|__\|\|__\| | | | | | | | | | |
|  |  |  | iii. Tubers *(lamang lupa katulad ng patatas, kamote)* | | | | | | | | | | | | | \|__\|\|__\| | | | | | | | | | |
|  |  |  | iv. Corn *(mais)* | | | | | | | | | | | | | \|__\|\|__\| | | | | | | | | | |
|  |  |  | v. Noodle/Pasta *(pansit)* | | | | | | | | | | | | | \|__\|\|__\| | | | | | | | | | |
|  |  | **3B.1b.** Beans, pulse, lentils, chickpeas, *tofu (Beans, munggo, kidney beans, garbanzos, tokwa)* | | | | | | | | | | | | | | \|__\|\|__\| | | | | | | | | | |
|  |  | **3B.1c.** Nuts and seeds *(mani, kasuy, pistachio, butong pakwan, atbp)* | | | | | | | | | | | | | | \|__\|\|__\| | | | | | | | | | |
|  |  | **3B.1d.** Dairy, milk, yogurt *(produktong gawa sa gatas tulad ng, keso, gatas, ice cream, yogurt)* | | | | | | | | | | | | | | \|__\|\|__\| | | | | | | | | | |
|  |  | **3B.1e.** | i. Meat (karne ng baboy at baka) | | | | | | | | | | | | | \|__\|\|__\| | | | | | | | | | |
|  |  |  | ii. Poultry (karne ng manok) | | | | | | | | | | | | | \|__\|\|__\| | | | | | | | | | |
|  |  |  | iii. Fish (Isda) | | | | | | | | | | | | | \|__\|\|__\| | | | | | | | | | |
|  |  |  | iv. Seafood (hipon, alimasag, tahong, atbp) | | | | | | | | | | | | | \|__\|\|__\| | | | | | | | | | |
|  |  |  | v. Organ meats (lamang loob) | | | | | | | | | | | | | \|__\|\|__\| | | | | | | | | | |
|  |  | **3B.1f.** Eggs *(itlog)* | | | | | | | | | | | | | | \|__\|\|__\| | | | | | | | | | |
|  |  | **3B.1g.** Dark green leafy vegetables *(berde at madahon na gulay)* | | | | | | | | | | | | | | \|__\|\|__\| | | | | | | | | | |
|  |  | **3B.1h.** Other vitamin A rich fruits and vegetables (carrot, pumpkin, orange sweet potato, mango, papaya, dark green leafy vegetables, long beans) *(Iba pang prutas at gulay na mayaman sa bitamina A)* | | | | | | | | | | | | | | \|__\|\|__\| | | | | | | | | | |
|  |  | **3B.1i.** Other vegetables (eggplant, onion, cucumbers, tomatoes) *(iba pang gulay)* | | | | | | | | | | | | | | \|__\|\|__\| | | | | | | | | | |
|  |  | **3B.1j.** Other fruits (banana, apples, pineapple, watermelon) *(iba pang prutas)* | | | | | | | | | | | | | | \|__\|\|__\| | | | | | | | | | |
|  |  | **3B.1k.** Sugary foods: chocolate bars, candy bars, candies, sugar-coated food, ice cream, honey, jam, candy, biscuits, pastries, cakes and other sweet products *(matatamis na pagkain)* | | | | | | | | | | | | | | \|__\|\|__\| | | | | | | | | | |
|  |  | **3B.1l.** Sugary drinks (carbonated soft drink, sweetened tea, sweetened coffee *(Matatamis na inumin)* | | | | | | | | | | | | | | \|__\|\|__\| | | | | | | | | | |
|  |  | **3B.1m.** Savory and fried snacks (street foods) *(malinamnam at pritong pagkain katulad ng kwek-kwek, fishball, turon, mga inihaw, atbp)* | | | | | | | | | | | | | | \|__\|\|__\| | | | | | | | | | |
|  |  | **3B.1n.** Others (i.e. chichirya/mantika/asin bilang ulam), specify: ______________ | | | | | | | | | | | | | |  | | | | | | | | | |
| **3B.2** | | Has your child’s eating consumption patterns (related to variations or types of food consumed) change because of Covid-19 pandemic?  *Nagbago ba ang pagkain ni ____ dahil sa covid19 pandemic?* | | | | | | | | | 1=Yes, increase consumption  *(Oo, tumaas ang pag konsumo)*  2=Yes, decrease consumption  *(Oo, bumaba ang pag konsumo)*  3=No change *(Walang pag babago)* | | | | | | | | | | | | | | \|__\| |
| **3B.3** | | Has your child’s food frequency change because of Covid-19 pandemic?  *Nagbago ba ang dalas ng pagkain ni _____ dahil sa covid 19?* | | | | | | | | | 1=Yes, increase frequency  *(Oo, mas dumalas ang pag kain)*  2=Yes, decrease frequency  *(Oo, nabawasan ang dalas ng pag kain)*  3=No change *(Walang pag babago)* | | | | | | | | | | | | | | \|__\| |
| **SECTION 4: ILLNESS AND CARE FOR 0-59 MONTHS CHILDREN (3 QUESTIONS)** | | | | | | | | | | | | | | | | | | | | | | | | | |
| **4.1** | | Has (*name*) had diarrhoea (loose watery stools 3 or more times in a day) in the last two weeks?  *Nagkaroon po ng diarrhea or madalas na pagdumi (tatlo or higit pa sa isang araw si ______ sa nakaraang dalawang lingo?* | | | | | | 0=No *(Hindi)* => **Proceed to Section 5**  1=Yes *(Oo)*  98=Do not know *(Hindi alam)* | | | | | | | | | | | | | | | | \|__\|\|__\| | |
| **4.2** | | Did you seek advice or treatment for the illness outside the home?  *Kumonsulta po ba kayo para sa sakit ni (pangalan) sa labas ng inyong bahay? Kung oo, kanino or saan?* | | | | | | **0**= No => Proceed to 4.3  1= Yes, Public Health facility *(pampublikong health facility)*  2= Yes, Private clinic *(pribadong klinik)*  3= Yes, Traditional healers *(e.g. albularlyo)*  4= Yes, Herbal stores/ Herbalists  5= Yes, Drugs store/pharmacy  6= Yes, Friends/relatives *(kaibigan/kamag anak)*  7= Yes, Others, specify __________________  **98** = Don’t know *(Hindi alam)* | | | | | | | | | | | | | | | | \|__\|\|__\| | |
| **4.3** | | What was the main reason for not seeking health care?  *Bakit hindi po kayo kumonsulta sa health care?*  [DO NOT READ OUT THE RESPONSE OPTION. SELECT THE RESPONSE OPTION THAT BEST FITS THE INFORMATION PROVIDED BY THE RESPONDENT] | | | | | | 0=risk of getting C19 *(banta na pag kakaroon ng Covid)*  1=lack of money *(kakulangan sa pera)*  2=absence of health services / hospitals, health centres are closed *(walang serbisyong pangkalusugan/sarado ang hospital at health center)*  3=no caretaker to take the child to the health facility *(walang mag dadala sa bata sa health facility)*  4=health facility too far away *(masyadong malayo ang health facility)*  5=movement restrictions *(pagbabawal sa pag labas at pag punta sa ibang lugar)*  6=denied access because it’s out of capacity *(hindi tinanggap dahil sa puno na ang health facility)*  7=all members of the family too sick to move *(lahat ng miyembro ng pamilya ay may sakit para madala ang bata sa health facility)*  8=others, specify _________________ | | | | | | | | | | | | | | | | \|__\| | |
| **SECTION 5 –FOOD PURCHASED FOR 0-59 MONTHS CHILDREN (2 QUESTIONS)** | | | | | | | | | | | | | | | | | | | | | | | | | |
| **5.1** | | Yesterday, did you purchase prepared meal for your child?  *Bumili po ba kayo ng lutong pagkain para sa inyong anak kahapon?* | | | | | | 0=No *(Hindi)*  1=Yes *(Oo)*  98=Do not know *(Hindi alam)* | | | | | | | | | \|__\|\|__\| | | | | | | | | |
| **5.2** | | Has the level of spending changed because of Covid 19 pandemic?  *Nagbago po ba ang pag gastos ninyo sa bahay ng dahil covid19 pandemic?* | | | | | | 0=No *(Hindi)*  1=Yes, increased spending *(Oo, tumaas ang pag gastos)*  2=Yes, decreased spending  *(Oo, nabawasan ang pag gastos)*  98=Do not know *(Hindi alam)* | | | | | | | | | \|__\|\|__\| | | | | | | | | |

READ TO THE RESPONDENT:

Thank you very much for the interview. As a token of appreciation, we will now transfer [amount] to this phone number. Have a great day.

Maraming salamat po sa inyong partisipasyon sa aming panayam. Bilang aming pasasalamat, aming bibigyan ng [AMOUNT] load ang numerong ito. Magandang Araw po!

Supplementary Material 2

1. Wasting, stunting and underweight are included in case anthropometric measurements can be collected in a follow-up survey. [↑](#footnote-ref-2)
