# Supplementary material for: Urban nutrition situation in the slums of three cities in Asia during the COVID‐19 pandemic
Source: Matern Child Nutr. 2023 Oct 9;22(1):e13543. doi: 10.1111/mcn.13543 (PMC12647976; doi:10.1111/mcn.13543)
Supplement: Supplementary file 2 — Supporting information. [file MCN-22-e13543-s001.docx]

**Supplementary Material 2**

Child and Maternal Nutrition, Market Access and COVID-19 Impact Indicators

1. Maternal and Child Nutrition

Table 1: Maternal and Child Nutrition Indicators

| Theme | Indicator (SDGs or core indicators in bold) | Description |
| --- | --- | --- |
| Food security | Food Consumption Score simplified | A composite score based on dietary diversity, food frequency, and relative nutritional importance of different food groups |
|  | Food Insecurity Experience Scale (FIES) | Per cent of the households experiencing moderate or severe food insecurity; and Per cent of the households experiencing severe food insecurity |
| Nutrition | Minimum dietary diversity for women index (MDD-W) | Per cent of women who consumed at least 5 out of 10 defined food groups the previous day or night |
|  | Timely initiation of breastfeeding | Per cent of women with a live birth in the 2 years preceding the survey that put the newborn infant to the breast within 1 hour of birth |
|  | Proportion of children exclusively breastfed until 6 months of age | Per cent of infants aged 0–5 months who were fed exclusively with breast milk during the entire day prior to interview. Exclusive breastfeeding means the baby has not received any other fluids (including water) or foods, with the exception of oral rehydration solution, drops and syrups (vitamins, minerals, medicines) |
|  | Minimum dietary diversity (MDD) | Per cent of children 6–23 months of age who receive foods from four or more food groups |
|  | Minimum Meal Frequency (MMF) | Per cent of breastfed and non-breastfed children 6–23 months of age, who receive solid, semi-solid, or soft foods (but also including milk feeds for non-breastfed children) the minimum number of times or more |
|  | Minimum Acceptable Diet (MAD) | Proportion of children 6-23 months of age who receive a minimum acceptable diet (apart from breastmilk) |

1. COVID-19 Impact Index Indicator

The impact index (**Fig. 1**) was determined based on the household’s response to the pandemic, i.e., shock severity and the livelihood based coping strategies detailed below.

1. **COVID-19 shock severity categories**
2. Little Shock: no concerns, disruption of educational institutes, getting sick, travel restrictions. “Other” concerns related to travel, internet for education, crime, people don't believe covid-19, buying water, news about covid-19 cause anxiety, Indonesian economy crash, worried not using mask.
3. Medium Shock: lack of work, disruption of livelihood source. “Other” concerns related to lack of work.
4. High Shock: shortage of food, increase in food prices, shortage of medicine, disruption of medical service. “Other” concerns related to disruption of medical service, paying the rent, daily need purchase (milk, diapers, etc.)
5. **Livelihood-Based Coping Strategies (LBCS) score categories**
6. LBCS = 4 / Emergency coping strategies: such as consuming seed stock held for the next season, selling house or land or last female animal, begging, or pawning house, land certificate or deed.

Did anyone in household have to engage in sold house or land?

Did anyone in household have to engage in begging?

Did anyone in household have to engage in pawn house/land certificate or deed?

1. LBCS = 3 / Crisis coping strategies: such as selling household or productive assets, or withdrawing children from school, taking on informal debt with interest.

Did anyone in household have to engage in sold productive assets or means of transport?

Did anyone in household have to engage in withdrew children from school?

Did anyone in household have to engage in informal debt with interest?

1. LBCS = 2 / Stress coping strategies: such as borrowing money, purchase food on credit, spent savings, selling household goods, pawning household assets like radio, furniture, television, jewellery, reducing non-food expenses on health and education, spending savings, borrowing money or food from a formal lender or bank.

Did anyone in household have to engage in sold household assets/goods?

Did anyone in household have to engage in reduced non-food expenses on health & education?

Did anyone in household have to engage in spent savings?

Did anyone in household have to engage in borrowed money/food from a formal lender/bank

Did anyone in household have to engage in informal debt without interest?

Did anyone in household have to engage in pawn household assets/goods?

1. LBCS = 1 / Not engaged in any such coping activities

Did anyone in household have no engage in any activities?

1. LBCS = 0 / None of the above

|  |  | COVID-19 SHOCK SEVERITY | | |
| --- | --- | --- | --- | --- |
|  |  | Little Shock | Medium Shock | High Shock |
| LIVELIHOOD BASED COPING STRATEGY (LBCS) SCORE | No Coping Strategy | **LOW** | **MODERATE** | **SEVERE** |
|  | Stress Coping | **MODERATE** | **MODERATE** | **SEVERE** |
|  | Crisis Coping | **SEVERE** | **SEVERE** | **SEVERE** |
|  | Emergency Coping | **SEVERE** | **SEVERE** | **SEVERE** |

Figure 1: COVID-19 Impact Index

1. Market Access Indicators

Table 3: Reduced physical and financial access to food

| **Reduced financial access** | **Yes if at least one of these conditions** |
| --- | --- |
| Substitute the usual food items with other cheaper food items | |
| Buying less of this food because reduced purchasing power/increasing prices | |
| Stopped buying because no money available to buy | |
| **Reduced physical access** | **Yes if at least one of these conditions:** |
| Buying less of this food because reduced access to functioning/open market facilities (physical access problem) | |
| Stopped buying because not available in the market | |
